# Facilitators and barriers to blood and blood product accessibility and use in sub-Saharan Africa: a systematic review

**DOI:** 10.3389/frhs.2026.1837188

**Published:** 2026-06-12

**Authors:** Adaeze Oreh, Kobi Vanessa Ajayi, Henshaw Uchechi Okoroiwu, Uchejeso Mark Obeta, Chioma Maduoma, Shirley Owusu-Ofori, Maarten Postma, Theresa Nwagha, Marinus van Hulst

**Affiliations:** 1 Faculty of Clinical Sciences, Pamo University of Medical Sciences, Port Harcourt, Nigeria; 2 Department of Health Sciences, University Medical Center Groningen, University of Groningen, Groningen, Netherlands; 3 Department of Health Behavior, School of Public Health, Texas A&M University, College Station, TX, United States; 4 Medical Laboratory Science Department, David Umahi Federal University of Health Sciences, Uburu, Nigeria; 5 Department of Medical Laboratory Management, Federal College of Medical Laboratory Science & Technology, Jos, Nigeri; 6 Department of Urgent Care, UC Urgent Care, Union City, GA, United States; 7 Office of the National Coordinator, National Blood Service, Accra, Ghana; 8 Center of Excellence for Pharmaceutical Care Innovation, Universitas Padjadjaran, Bandung, Indonesia; 9 Department of Hematology, University of Nigeria Teaching Hospital, Enugu, Nigeria; 10 Department of Clinical Pharmacy and Toxicology, Martini Hospital, Groningen, Netherlands

**Keywords:** access to healthcare, blood, blood products, sub-Saharan Africa, WHO health systems framework building blocks, whole blood

## Abstract

**Background:**

Access to safe blood and blood products is a global health priority, requiring concerted public health efforts to ensure universal access to reduce the global disease burden. However, there are inequities in access to safe and adequate blood and blood products in sub-Saharan Africa (SSA), leading to marked challenges in meeting the clinical needs of patients with diverse pathologies. This study used the WHO health system building blocks to systematically synthesize the literature to understand the barriers to blood and blood product usage in SSA.

**Methods:**

We used the PRISMA guideline to systematically search relevant articles using six electronic databases: Web of Science, MEDLINE, PubMed, PscyInfo, Google Scholar, and Global Health databases between 2005 and 2023. The risk of bias for included studies was assessed using a modified Joanna Briggs Checklist. Inductive thematic analysis was performed to thematize the extracted data based on the WHO health system building blocks.

**Results:**

Sixty-five studies representing eighteen countries in SSA were included for review. Barriers included transfusion delays, low transfusion rates, inappropriate practices, untimely referrals, blood stock-outs, poor adherence to guidelines, lack of standardized protocols, poor transfusion management systems, poor leadership/governance, and health workforce shortages. Facilitators included drone technology use, prompt referrals, appropriate transfusions, availability of skilled workforce, health information systems, and effective leadership and governance.

**Conclusions:**

Numerous barriers to blood and blood product access in SSA exist. Addressing service delivery barriers such as transfusion delays, product stock-outs, and ineffective governance of transfusion systems would be vital. Further, innovative technology, boosting referral systems, and practicing appropriate use of blood would engender sustainable access to safe blood services in SSA. Innovations such as the successful deployment of drone technology in Rwanda are worth emulating and scaling across the continent. Also, financing and effective leadership should be further researched as potential facilitators of safe blood accessibility and use in Africa.

**Systematic Review Registration:**

https://www.crd.york.ac.uk/PROSPERO/view/CRD42023434335.

## Background

According to the World Health Organization (WHO) Model List of Essential Medicines, whole blood, and blood products such as red blood cells, platelets, and fresh-frozen plasma are critical to meet the healthcare needs of populations (1). As a result, access to safe blood and blood products is a global health priority, and concerted public health efforts are needed to ensure universal access to reduce the global disease burden. However, there are disparities in access to safe and adequate volumes of blood and blood products in low- and middle-income countries (LMICs), particularly in sub-Saharan Africa (SSA). Therefore, marked challenges are experienced in meeting the clinical needs of diverse pathologies. For example, in 2022, a WHO African Region survey report revealed that 5.2 units of blood per 1000 people were donated in many African countries, far below the recommended ten units/1,000 population (2). From the data, only six African countries met the WHO targets. Thus leading to unacceptably high preventable mortalities and morbidities in the region compared to high-income countries (2–5).

Although numerous and complicated structural and patient-level factors are associated with the shortage of blood and blood products in SSA (4, 5), research has demonstrated poor blood transfusion practices as an important cause of hospital admissions and mortality in the region (6–8). Notably, lack of or delayed blood transfusions in SSA is associated with increased pediatric malaria-related mortalities (6–8) and severe perinatal hemorrhage, contributing to over 72% of maternal deaths (9, 10). Consequently, SSA accounts for the highest number of under-five children and maternal deaths worldwide—74 under-five deaths per 1,000 live births in SSA, 14 times higher than in developed nations, and a maternal mortality ratio of 542 (UI 498–649) maternal deaths per 1,00 000 live births, accounting for almost 66% of global maternal deaths, respectively (2, 11, 12). Timely transfusion in severely anemic children and following peripartum hemorrhage has been shown to maximize the chances of survival (9, 13, 14). Unsurprisingly, blood transfusion in SSA follows a similar pattern, with higher transfusion rates in pediatric and obstetric settings than in other settings. Furthermore, transfusion-transmissible infections (TTI) such as HIV, hepatitis B, and hepatitis C continue to pose real quality assurance and management concerns because they are endemic in the region (2). Despite national transfusion policies and guidelines supporting studies that suggest slow and steady progress in reducing TTIs, there is vast room for improvement in blood donor selection, recruitment, retention, testing, and screening (3, 5, 15). To illustrate this, median blood discard rates due to TTI reactivity (the number one reason for discarding blood in African countries) were 4–5 times higher in low-income countries (LICs) and LMICs. Of the discarded blood reported by WHO, (9.6% in low-income countries, and 8.0% in lower-middle-income countries), more than half (5.8% in LICs and 4.4% in LMICs) were discarded primarily because of TTIs (16).

Various clinical indications in emergency and non-emergency situations drive the demand for blood or hamper the supply by reducing donations. For instance, public health emergencies such as the Ebola outbreaks and the COVID-19 pandemic, which had a significant impact on SSA because of existing deficiencies in the healthcare system, brought to the fore the enormous challenges to maintain the capacity to ensure safe and reliable blood supply on the continent (15, 17). However, considering that a large part of the region is plagued with a lack of infrastructure, persistently depleting skilled health workforce, financial challenges, and many other challenges, establishing a robust infrastructure for universal access to blood and blood products requires strong commitment from diverse stakeholders at all levels, most notably government policies and legislation.

Due to the complexities of ensuring safe and reliable blood and blood products in SSA, the WHO Health Systems Framework could provide a basis for understanding the challenges in achieving safe blood targets and objectives outlined in the WHO Action Framework (18, 19). This systems framework includes 1) service delivery, 2) health workforce, 3) health information systems, 4) access to essential medicines, 5), financing, and 6) leadership/governance. The WHO Health Systems Framework catalyzes health system strengthening in diverse ways. It has been used to evaluate the performance of healthcare facilities (20), assess disease control programs (21), evaluate public health emergency preparedness (22), measure community health services and planning (23), and even management of some health conditions (24). The performance of these building blocks is therefore crucial to strengthening blood transfusion systems, particularly in SSA, to improve safe blood availability. This would have implications for health indices such as maternal, newborn, infant, and child mortality, which are linked to poor blood infrastructure and supply chains.

This study sought to 1) investigate blood transfusion practices and patterns in SSA, 2) evaluate the indications for blood and blood product use, and 3) use the WHO building blocks to assess barriers and facilitators of blood and blood product use. By doing so, we aim to contribute to understanding how further improvements in blood transfusion practices in SSA could be achieved.

## Methods

This systematic review used the Preferred Reporting Items for Systematic Reviews and Meta-Analyses (PRISMA) reporting guidelines and checklist. The study protocol was developed and registered in the National Institute for Health and Care Research with the PROSPERO registration number CRD42023434335.

### Data source, search strategy, and study selection

Literature was searched from Web of Science, MEDLINE, PubMed, PscyInfo, Google Scholar, and Global Health databases. The search strategy was limited to articles published in English between 2005 and September 2023. An updated search using the same keywords was conducted between 2023 and February 2026. The keywords used included: “blood transfusion,” “blood products,” “plasma,” “red blood cells,” “platelets,” “fresh frozen plasma,” “cryoprecipitate,” “access,” “utilization,” and all countries within the sub-Saharan African region (see Supplementary Appendix 1). Inclusion criteria such as peer-reviewed research articles published in English Language, reporting blood and blood product transfusion, access, or utilization in sub-Saharan Africa, including all study designs (i.e., quantitative or qualitative), and addressing any patient demographic or health condition related to the study question were used. See Supplementary Appendix 1 for full information.

### Data synthesis and analysis

Datapoints and headings were extracted based on their relevance to the review's objectives: author year, study setting (country), study design, characteristics of participants, type of blood or blood products reported, use of blood or blood products, and main findings. The WHO Health System Building Framework was also used to characterize observed barriers and enablers or facilitators of blood and blood product use. Some articles reported barriers and facilitators of blood and blood product use or access. As a result, an article may be grouped under both categories. Further, we empirically ordered articles into the six building blocks based on the alignment between the reported findings and the blocks. For example, an article would be categorized under “workforce” if it reports positive or negative sentiments about healthcare workers. Concerning studies that included SSA and non-SSA countries, we reported and analyzed only the disaggregated findings for SSA. However, we reported the comparative findings between the countries where applicable to provide additional, comprehensive insights about the results. Consequently, the findings were summarized and organized in themes using qualitative narrative inductive thematic analysis. Using thematic analysis allowed for the identification and interpretation of salient data features that corresponded with the research objectives and questions.

### Quality appraisal

Two independent reviewers appraised each study's risk bias using five Joanna Briggs Institute (JBI) Checklists (25): analytical cross-sectional, cohort, case-control studies, case reports, and qualitative research checklists to appraise each study's risk of bias. Another reviewer acted as a mediator to resolve discrepancies. Because the studies in this review comprised various study designs, we combined and modified the above-mentioned JBI checklists by merging relevant criteria into a comprehensive tool. The quality score for each included study was calculated and then interpreted as yes, no, or not applicable (Supplementary Appendix 2).

## Results

The study aimed to investigate blood transfusion practices and patterns, evaluate the indications for blood and blood product use, and assess barriers and facilitators of blood and blood product usage in SSA using the WHO health system framework.

### Screening result

**Figure 1:**
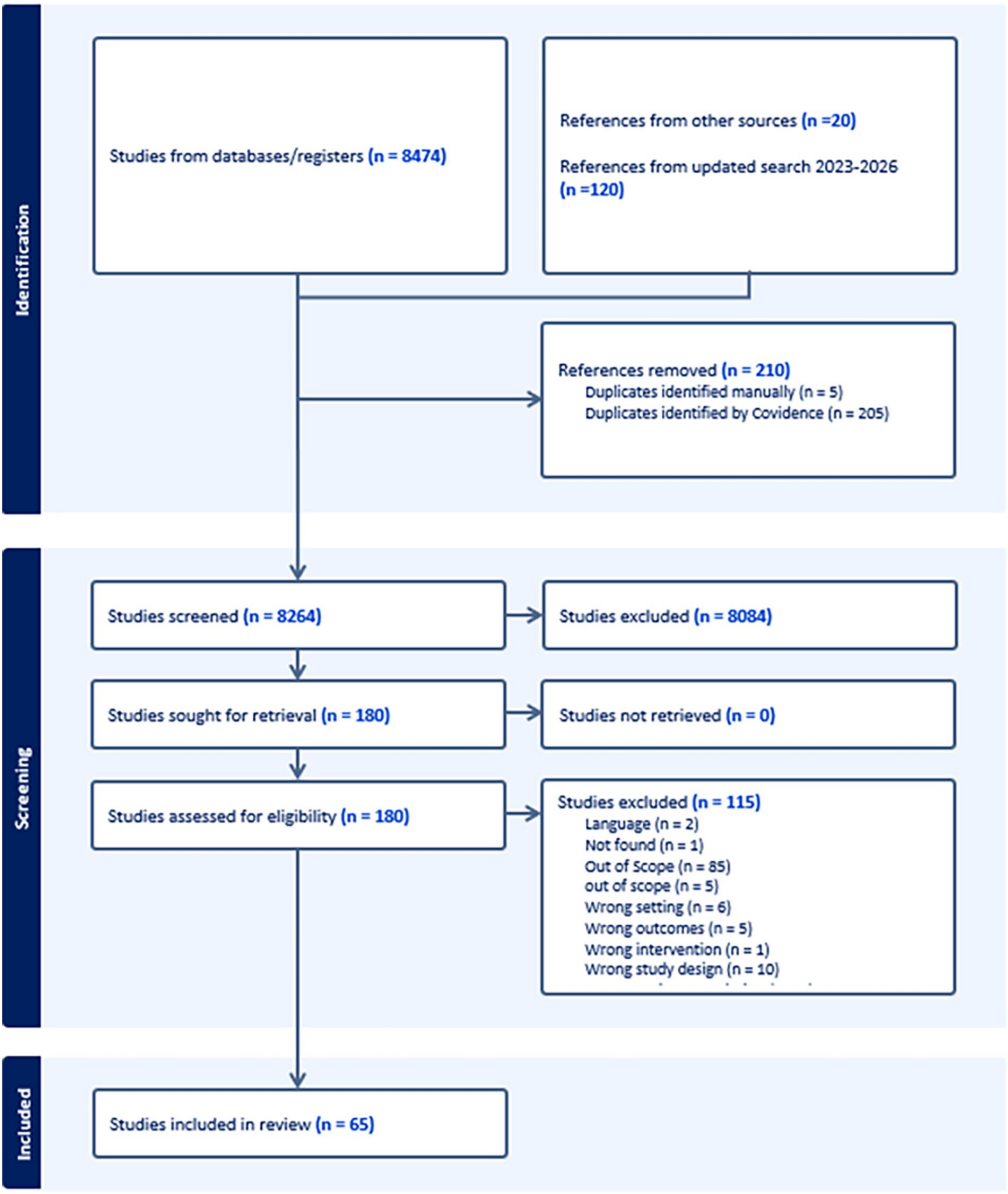
PRISMA flow diagram detailing the database searches, the number of studies screened, and the full texts retrieved and included for review.

An initial search yielded 8,474 studies identified from the included electronic databases. After duplicates (210) were excluded from the search results, 8,264 articles underwent title and abstract screening. Of these, only 180 articles met the criteria for full-text screening, after which 65 studies were found to be eligible for analysis. The PRISMA flow diagram, Figure 1, shows a detailed breakdown of the articles included for review.

### Study characteristics

Eighteen (*n* = 23) SSA countries were represented in this study. Most were from Nigeria (*n* = 22/65; 33%) (26–47), followed by Ethiopia, South Africa, and Kenya had (*n* = 7/65; 10%) studies each 7, 30, 44, 48–58. Malawi, Tanzania, and Ethiopia had (*n* = 5/65; 7%). Other countries represented included Ghana, Sudan, Rwanda, Zimbabwe, Mozambique, Burkina Faso, Senegal, Mali, Democratic Republic of Congo, Namibia, Lesotho, The Gambia, Burundi, Liberia, Eswatini, and Botswana (30, 44, 59–64).

Almost all (*n* = 59/65; 90%) of the studies occurred in healthcare facilities (Table 1). These facilities comprised private, public, and faith-based organizations (7, 13, 26–29, 31–39, 42–50, 52, 54–60, 62–87). Three (*n* = 3/65; 4%) (51, 61, 88) used data from national blood transfusion banks. Other studies included participants from the community where the study was conducted, and only one (*n* = 1/65, 1%) study was conducted in a community-based setting (86). One study was conducted at a scientific conference (*n* = 1/65, 1% ) (41).

**Table 1:** Characteristics of the included studies.

Author/Year	Country	Study Type	Study Setting	Population Description	Sample Size	Study Objectives
Ahmed et al. (2019) (70)	Eastern Sudan	Cross-sectional study	Tertiary hospital	Children below 18 years who presented at the outpatient department and emergency room with an indication for blood transfusion between August 1, 2017, and March 31, 2018.	1,800	The study aimed to provide information on the rate of anemic children who received a blood transfusion and to describe the indications, outcome, time needed to transfuse, and mortality following transfusion.
Akech et al. (2008) (55)	Kenya	Prospective observational study	Kenya Medical Research Institute at a District Hospital.	Children with severe malaria and metabolic acidosis.	213 severe malaria survivors of the 241 cohort of children admitted with severe malaria complicated by metabolic acidosis.	To assess the outcome and hematological recovery of children with severe malaria where transfusion practice compiles with WHO recommendations.
Akingbola & Bello, (2016) (27)	Nigeria	Cross-sectional	Tertiary hospital	Obstetric patients between January 2009 and December 2014.	565	To explore transfusion rate, identify the common indications for transfusion, and review blood products administration in obstetrics patients.
Akinlusi et al. (2018) (34)	Nigeria	Prospective observational study	Obstetric unit at a university teaching hospital	Pregnant women who attended the maternity unit for cesarean deliveries between January 1st and December 31st, 2011.	906	To identify and evaluate the risk factors for blood transfusion among women with cesarean deliveries.
Akoko & Joseph, (2015) (83)	Tanzania	Descriptive cross-sectional	National hospital	Patients who underwent elective surgical surgery between March and October 2013.	445 patients	To evaluate the efficiency of blood ordering and its subsequent utilization among elective adult urological and general surgical patients and to recommend a maximum surgical blood ordering scheme for a national hospital.
Akpa et al. (2022) (33)	Nigeria	Matched case-control	Health facilities comprising teaching hospitals and clinics.	Patients with or without sickle cell disease	2,034	To investigate the demographic, clinical, and environmental or behavioral factors associated with selected transfusion transmissible infections in sickle cell disease (SCD).
Aliyu et al. (2017) (38)	Nigeria	Retrospective study	Hospital	Patients attending a hospital between January 1, 2011 and December 31, 2011.	1,958	To determine the blood request pattern and, if any, periodic variation in demand for blood transfusion.
Akwiwu et al. (2024) (47)	Nigeria	cross-sectional	Healthcare facility	Women with breast cancer	55	To evaluate the blood transfusion supportive care rendered to breast cancer patients
Amadi et al. (2023) (29)	Nigeria	Prospective observational study	University Teaching Hospital	All women in the obstetrics and gynecology department who received blood transfusions between July 1 and December 31, 2021.	1,000	To evaluate the indications and rate of blood transfusion, local use pattern, and variability of blood type transfused based on the recipient's blood type.
Assennato et al. (2018) (81)	Ghana	Retrospective observational study	A teaching hospital	Female children aged 5 or younger who had received a single transfusion of male whole blood for severe anemia related to malaria. Female patients transfused with female blood or not transfused at all and male patients transfused with male blood.	27	to assess transfusion-related microchimerism (TA-Mc) in young Ghanaian female recipients of male whole blood transfusions
Asuquo et al. (2025) (45)	Nigeria	Cross-sectional	Healthcare facility	Patients with sickle cell diseases who had one or more pregnancies and had their diagnosis confirmed by hemoglobin (Hb) electrophoresis.	40 pregnant women with sickle cell diseases	To determine the maternal outcomes of pregnant women living with sickle cell diseases.
Bassey et al. (2024) (46)	Nigeria	Cross-sectional	Healthcare facility	Children with cancer receiving chemotherapy	32	To assess the transfusion usage of the various blood components for childhood cancer patients in the Pediatric Oncology unit of a tertiary hospital.
Birhan & Asfaw, (2019) (75)	Ethiopia	Cross-sectional	Hospital-based	Patients who had surgical procedures between February 1, 2017, to September 30, 2017	387	To determine the factors associated with blood transfusion in a specialized Hospital in Addis Ababa, Ethiopia.
Bloch et al. (2018) (49)	South Africa	Case-Control	Four hospitals	Women with pregnancies of at least 26 weeks gestation. Stillborn, early neonatal deaths, vaginal delivery, and births by cesarean section between 31st March 2014 to 31st October 2015. Cases comprised women who were transfused 48 h before or after delivery. Controls included a random sample of non-transfused deliveries.	1,200 cases and 2,434 controls.	To determine the risk factors for peripartum blood transfusion among peripartum women with very high HIV prevalence.
Bolton et al. (2021) (51)	South Africa	retrospective observational study	South African National Blood Service (SANBS)	RBC product requests for both public and private patients submitted to SANBS blood banks between 1 January 2014 and 31 March 2019	Not described	1) To investigate red blood cell product utilization trends between public and private healthcare sectors serviced by the South African National Bank Services. 2) To understand temporal sector-specific patterns of red blood cell utilization.
Bugge et al. (2012)	Malawi	cross-sectional study	Hospital	Patients needing a blood transfusion.	104	Investigate the utilization of blood transfusion services within a hospital in Malawi and evaluate the adherence to WHO guidelines regarding the quality of these transfusion services.
Chansa et al. (2013)	Zambia	Case report	Hospital	An unconscious 2-year and 7-month-old child who was admitted to the emergency unit due to hypovolemic shock and anemia due to severe epistaxis.	1	This is a case report of a 31-month-old child presenting with hypovolemic shock and anemia due to severe epistaxis, who received an IO blood transfusion through an 18-gauge hypodermic needle.
Checkley et al., (2019) (86)	Uganda	Mixed-method, Prospective cross-sectional	Community members and data from a regional referral hospital.	Community members and health professionals between June 2017 and August 2017	82 community members, 20 health professionals, and 373 blood transfusion records	The study aimed to: 1) understand community member's knowledge of attitudes towards and practices of blood donation, 2) understand the health professionals' attitudes towards and practices of blood transfusion, 3) describe the distribution of patients receiving blood products, and 4) determine the process mapping of a single blood unit from donation to transfusion.
Chiabi et al. (2024)	Cameroon	Cohort	Healthcare facility	Children with cancer receiving chemotherapy	6,599	To determine the prevalence of pediatric blood transfusions at the one regional hospital, and identify the profile and outcome of the transfused children.
Dhabangi et al. (2019) (76)	Uganda	Qualitative study	3 regional referral hospitals	Caregivers of recently transfused children with severe anemia and community members.	16 interviews and six focus group discussions with six to eight participants each.	To describe the community perceptions of blood transfusion for pediatric severe anemia in Uganda.
Diaku-Akinwumi et al. (2016)	Nigeria	Cross-sectional	Public hospitals in Nigeria	Public secondary and tertiary hospitals in Nigeria. Data was collected between March and August 2014.	31 providers from 31 hospitals	To document the level of care provided in specialist hospitals with dedicated sickle cell clinics in Nigeria and their ability to provide safe blood transfusion as part of standard-of-care and to identify associated challenges.
Drammeh et al. (2018) (67)	Tanzania	Prospective cross-sectional	Private, government, and faith-based hospitals	Blood transfusion in patients at the hospitals between June 17 and September 27, 2013.	42	The study aimed to describe the clinical indications for transfusion in Tanzania by assessing the current ordering and utilization of blood in public and private transfusion hospitals.
Efobi et al. (2021) (21)	Nigeria	Cross-sectional	Scientific Conference	Physicians attending a scientific conference	69 physicians	To assess the knowledge and practice of safe blood transfusion in MTPs by physicians.
Eyelade et al. (2015) (26)	Nigeria	Prospective cross-sectional study	University hospital.	Pregnant patients scheduled for emergency or elective cesarean section between March and August 2011.	706	To determine risk factors for blood transfusion in patients who had Caesarean delivery and to compare blood transfusion rates in HIV-negative patients with HIV-positive patients.
Fenta et al. (2024) (63)	Ethiopia	Cross-sectional	Healthcare setting	Hospitals, health centers, clinics, and health posts	632 facilities that provide blood transfusion services	To examine the service readiness in facilities offering blood transfusion and its determinants in Ethiopia
George et al. (2022) (71)	Uganda and Malawi	Secondary analysis of a randomized controlled trial	Three hospitals in Uganda and one hospital in Malawi	Children aged two months and 12 years were admitted for severe anemia (hemoglobin <6 g/dL) between September 17, 2014, and May 15, 2017.	3188	To examine the safety and efficacy of different pack types (whole blood vs. red cell concentrates) on hematological correction, re-transfusion, mortality, and readmission to hospital.
Gyedu et al. (2021) (74)	Ghana	Retrospective	Teaching hospital	Data from patients at a teaching hospital who presented with injuries requiring surgical intervention from January 2015 to December 2016.	1,116 patients	To determine the degree of appropriateness of blood transfusion among patients presenting with injuries that required surgical intervention at presentation to a tertiary hospital in Ghana.
Ijah et al. (2024) (40)	Nigeria	Retrospective study	One University Teaching Hospital	General surgery patients who underwent elective and emergency procedures.	78	To evaluate the intraoperative use of blood and blood products among General Surgery patients who underwent elective and emergency procedures.
Jacobs et al. (2023) (44)	Rwanda, Senegal, Sudan, Tanzania, The Gambia, Uganda, Zambia, Zimbabwe, Botswana, Burundi, Cameroon, Cote d'Ivore, Egypt, Eswatini, Ethiopia, Ghana, Kenya, Lesotho, Liberia, Malawi, Mozambique, Nigeria	Cross-sectional	Healthcare setting	Hospitals, Laboratory/clinic, national blood service	Individuals representing 137 unique institutions	To determine and describe the type of blood supply and blood bank/transfusion medicine services available at institutions across Africa.
Jatau et al. (2023)	Nigeria	Retrospective study	Hospital setting	Patient's blood transfusion request forms between January 2022 and December 2022.	8,548 forms	To assess the trend of blood transfusion requests and utilization and to identify areas of wastage.
Keating et al. (2021) (13)	Malawi	Retrospective cohort study	Hospital	Children admitted to the pediatric ward from September 2017 to September 2019.	1,761	To determine whether receiving blood transfusions was associated with lower mortality in children with severe anemia at different transfusion thresholds. The study also aimed to determine whether the association between transfusion and mortality differed between children with and without malaria.
Kintieti et al. (2026) (64)	Democratic Republic of Congo	Cohort	Healthcare facility	All patients aged 60 years or older who underwent anesthesia for orthopedic surgery	168	To evaluate the frequency and factors associated with transfusion in elderly orthopedic surgical patients.
Kiguli et al. (2015) (57)	Kenya, Tanzania, Uganda	Clinical trial data	Six hospitals across the three countries	Children between 60 days and 12 years presenting with severe febrile illness and clinical signs of impaired peripheral transfusion.	3,082	To report the prevalence, clinical features, and transfusion management of anemia in children with serious febrile illness and clinical signs of impaired peripheral perfusion.
Linstrom et al. (2024)	South Africa	cross-sectional study	Healthcare facility	Pregnant women	12,889 pregnant mothers	To describe hematological profiles of pregnant patients together with factors associated with high transfusion requirements in transfused obstetric patients.
Mafirakureva et al. (2015) (72)	Zimbabwe	Retrospective analysis	Four major hospitals comprising three public sector hospitals and one private hospital	All patients with blood transfusion receipts between January 1 and December 31, 2012.	1,793 patients	To describe the characteristics of blood transfusion recipients and the pattern of blood and blood component utilization.
Mafirakureva et al. (2016) (88)	Zimbabwe	Economic evaluation	Data from the National Blood Service Zimbabwe (NBSZ). NBSZ is responsible for blood supply to all private and public hospitals in Kenya.	Not described	70,834 donation visits in 2013 amounted to 67,440 visits accepted for donation.	To assess the unit costs and cost drivers for blood and blood components production at the National Blood Service Zimbabwe (NBSZ) using an activity-based costing (ABC) model.
Mandar et al. (2022) (68)	Eastern Sudan	Cross-sectional	Maternal hospital	Women who underwent emergency or elective cesarean delivery between March 1 and September 30, 2020.	539	To measure the prevalence of blood transfusion and its associated factors among women who delivered through cesarean birth.
Morris et al. (2019) (50)	South Africa	Cross-sectional	Data from three secondary-level hospitals without a blood bank and one tertiary hospital with a blood bank	Patients who received emergency blood from a blood fridge located in the emergency center at the three secondary-level hospitals. Patients from the tertiary hospital received emergency blood from an onsite 24-hour staffed blood bank.	210 patients	To assess the indications for which and locations where emergency blood was transfused from the stock, kept at the emergency centers of three secondary-level hospitals and one tertiary hospital.
Mumo et al., (2023) (56)	Kenya	observational study	Health facility	Residents of a county that has received and transfused at least one blood unit in emergencies between 2018 and 2021.	15 hospitals in the county that provide transfusion services	To assess travel time to hospitals offering blood transfusion services, spatial competition between these hospitals, and the spatial accessibility of blood transfusion services across the county.
Musa et al. (2014) (36)	Nigeria	Prospective study	Teaching hospital	Blood request forms and cross-match worksheets of a blood bank at a teaching hospital between June 1 and August 31, 2013.	986 patients	To evaluate the pattern of blood transfusion requests and utilization to determine transfusion practice.
Nabwera et al. (2016) (54)	Kenya	Prospective observational study	A general hospital.	Patients who requested blood transfusion 14 years and below between May 2005 and April 2007.	2,789	To describe clinical and laboratory practice and identify potential areas for intervention to improve the efficacy and safety of blood transfusion for children.
Natukunda et al. (2010) (84)	Uganda	Retrospective study	Regional Referral Hospital	Medical records of patients who received blood transfusions in 2008.	1,730	To assess the clinical transfusion practice at the facility.
Nisingizwe et al. (2022) (80)	Rwanda	Cross-sectional	Five facilities: referral, provincial, district and hospitals, health centers, and health posts.	District and provincial hospitals that received drone delivery between December 21, 2016, and Dec 22, 2018.	20 district and provincial hospitals	To investigate the impact of unmanned aerial vehicle (UAV) use on blood delivery times and blood product expirations in Rwanda.
Njolomole et al. (2022) (79)	Malawi	Qualitative	Two hospitals, central and district hospitals.	Diverse stakeholders working in the blood delivery pipeline, including policymakers, health professionals, patients, blood donors, and hospital administrative staff. The study was done between July 2020 to January 2021.	47	To understand the factors that affect timely and adequate access to blood and blood products for obstetric emergencies in 2 hospitals.
Nwafor et al. (2018) (32)	Nigeria	Retrospective	Hospital	Patients requiring open-heart surgery between March 2013 and February 2016.	102 patients	To review local experience in using blood and blood products during open heart surgery.
Okoroiwu & Okafor (2018) (35)	Nigeria	Retrospective	Teaching hospital	Patients record at the blood bank register between March 2016 and February 2017.	NA	Establish local use patterns of blood and blood products to manage patient needs effectively.
Olupot-Olupot et al. (2016)	Uganda	Randomized trial	Four hospitals in Uganda.	Children who registered at the trial with severe febrile illnesses.	318	To describe the prevalence and outcome of clinically reported dark urine potentially [blackwater fever (BWF)] among children.
Opoka et al. (2018) (69)	Uganda	Retrospective mixed method review	Children's ward at two regional referral hospitals.	Patients aged 0–5 years were diagnosed with severe anemia between June 2016 and May 2017.	2,775	To describe the extent of the appropriateness of the use of blood transfusion in children admitted with a diagnosis of severe anemia.
Oreh et al. (2022) (39)	Nigeria	Retrospective study	34 tertiary hospitals	Data of blood entries on a health information system compared between January to July 2019 (pre-COVID-19) and January to July 2020 (peri-COVID-19).		To investigate the effect of the COVID-19 pandemic on the number of blood donations and transfusions across tertiary hospitals and disaggregated by blood product type.
Patidar et al. (2022) (30)	Multiple countries: Democratic Republic of Congo, Ethiopia, Kenya, Morocco, Nigeria, and Rwanda.	Cross-sectional	International society of blood transfusion.	Data from members of the International Society of Blood Transfusion between October 2021 and November 2021.	54 respondents from 20 countries.	To assess the current status of infrastructure and resources in blood establishment and blood transfusion services in low-and-middle-income countries (LMICs) and to explore their challenges and plans for improvement.
Pitman et al. (2014)	Namibia	Retrospective	National database of blood banks in Namibia	Data from patients needing blood services between August 1, 2007, to July 31, 2011.	91,207	To evaluate indications for blood use in Namibia.
Ramtohul et al. (2022) (48)	South Africa	Prospective cross-sectional study	Three public hospitals	Obstetric patients with a gestational age greater than 26 weeks undergoing elective and emergency cesarean section deliver	1533	To investigate the prevalence and associated factors for blood transfusion among obstetric patients delivering through cesarean section.
Reggiani et al. 2019	Mozambique	Retrospective	A tertiary and teaching center	Data of patients in the pediatric emergency and neonatology unit between 2015 and 2016	1595 completed forms of patients	To analyze adherence to WHO indicators for transfusion requests and capacity to meet the demand in the pediatric emergency unit and to compare clinical and laboratory diagnoses of severe anemia.
Salami et al. (2022)	Nigeria	Retrospective study	University hospital	Obstetric patients who underwent cesarean section birth between 2016 and 2022	693	To identify the risk factors for blood transfusion in patients who had cesarean deliveries.
Sawadogo et al. (2020) (59)	Burkina Faso	Cros-sectional	Hospital	Children aged 0 to 14 were admitted to a pediatric ward between September and November 2016.	402	To assess the quality of transfusion requirements among children with severe malaria anemia.
Shari et al. (2017) (66)	Tanzania	Prospective observational study	Emergency department of a national hospital	Children under five years with anemia from August to September 2015.	257	To assess the burden of anemia in children admitted to an emergency unit to evaluate the emergency blood transfusion practices and outcomes of children with anemia.
Tadeu and Geelhoed, (2016) (73)	Mozambique	Qualitative	Three primary health facilities with and without a laboratory.	The blood service pipeline stakeholders include laboratory technicians, clinicians, community leaders, and patients.	57 focus group participants; the number of participants in the interviews was not reported.	To explore the role of laboratory services in primary healthcare concerning access, perceived service quality, and disease control.
Tewabe et al. (2022) (82)	Ethiopia	Cross-sectional	Hospital	Patients with data who received blood and blood component transfusion from May 1, 2021, to July 2021.	616	To assess transfusion utilization based on WHO guidelines and patient outcomes.
Thomas et al. (2017) (7)	Kenya	Retrospective	10 Hospitals nested in the Clinical Information Network in Kenya.	Pediatric nonsurgical admissions aged 1 month and above between September 2013 and March 2016.	53,174 children were admitted to the hospitals during the study period.	To examine the ability of clinicians to access blood when needed in Kenya after efforts to improve supply by exploring evidence of any delays in transfusion in Kenyan hospitals.
Tort et al. (2015) (60)	Senegal and Mali	Cluster randomized trial with 1-year baseline period, 2-year intervention period, and 1-year post-intervention period.	46 participating referral hospitals.	Mothers who delivered in the participating hospitals during the baseline period of the study between Sept 1, 2007 and Oct 30, 2011.	3,278	to identify factors associated with maternal death occurring during PPH in referral hospitals in Senegal and Mali, including women's characteristics, aspects of pregnancy and delivery, components of PPH management, and organizational characteristics of the hospitals.
Tsima et al. (2016) (62)	Botswana	Retrospective cross-sectional study	Four public hospitals	Admitted patients with a primary diagnosis of spontaneous or induced abortion between 1 January and 31 August 2–14.	619	To provide an overview of the present utilization of blood and blood components in the context of post-abortion care in Botswana
Uche et al. (2025) (43)	Nigeria	Cross-sectional	Healthcare setting	Blood banks within the healthcare institutions	13 healthcare institutions that provide blood transfusion service	To evaluate the blood transfusion practices in healthcare institutions across one Nigerian state, focusing on institutional characteristics, blood group distribution, blood component availability, and blood storage and cross-matching equipment.
Umar et al. (2024) (42)	Nigeria	Cross-sectional	Healthcare setting	Government tertiary hospital	50 Government tertiary hospitals	To determine blood donation practices, processing and utilization of blood components across government tertiary hospitals
Weeber et al. (2018) (52)	South Africa	Retrospective cross-sectional study	Hospital emergency center	Injured patients of all ages but not those with isolated burns or head injuries were triaged for care to the resuscitation area of the hospital's emergency center.	294	To investigate the amount of blood loss from serious injury concerning the availability of emergency blood products at a hospital with no on-site blood bank service.
Wentzel et al. (2019) (53)	South Africa	Retrospective study	National reference center for hereditary angioedema	Patients with acute swelling from 6 South African National Reference Centers for hereditary angioedema.	27	To evaluate the efficacy and safety of on-demand plasma treatment for acute hereditary angioedema.

Concerning study design, sixty (*n* = 60/65; 92%) (7, 13, 26–39, 41–72, 74, 75, 77, 78, 80–85, 87) employed quantitative methodologies consisting of cross-sectional studies, randomized control trials, case-control, and cohort studies, and one study used an economic evaluation methodology (*n* = 1/65, 1%) (88). Qualitative and mixed method studies were also included but represented only three (*n* = 3/65; 4%) and two (*n* = 2/65; 3%) (69, 73, 76, 79, 86), respectively. Methodological quality assessment of all included studies using the modified JBI Checklist indicated that most of the studies (92%) had a high rating across the JBI metrics suggesting that they showed methodological rigor. However, some articles were lacking in reporting key metrics or did not report them at all limiting the ability to assess the methodological rigor (See Supplementary Appendix 2).

### Blood transfusion practices and patterns

#### Blood and blood components reported

Overall, whole blood was more commonly ordered and used for blood transfusions. Of the sixty-five studies included for analysis, fifty (*n* = 50/65; 76%) utilized whole blood (7, 13, 26–31, 33–39, 42–44, 46–48, 50, 54–62, 65–72, 74, 78, 79, 81–88). Conversely, slightly below half (*n* = 31/65; 47%) of the studies reported blood components. These products included packed red cells, fresh frozen plasma, cryoprecipitate, platelets, red blood cells concentrate, platelet-rich plasma, plasma concentrate, allogeneic red cells, true packed red blood cells, leukocyte-depleted cells, leuko-reduced blood components, washed red cells, and modified plasma. Fresh frozen plasma was the most utilized component (*n* = 20/65; 30%). At least nineteen studies (*n* = 25/65; 38%) reported using whole blood and components concurrently. (Table 2)

**Table 2:** Prevalence, type of transfusion, and health domain reported.

Author/Year	Country	Blood Transfused	Blood Products Transfused	Prevalence of Transfusion	Health Domain	Type of Care	Main Findings
Ahmed et al. 2019 (70)	Eastern Sudan	Whole blood 300 (13.6) anemic children under 5 received transfusion. 513 (28.5%) were anemic, of which 141 (7.8%) had severe anemia. 285 (95.0%) improved after transfusion 9 (3.0%) were discharged against medical advice. 6 (2.0%) died.	NA	13.60%	Pediatric	Multiple	The percentage of blood transfusions indications include, sickle cell disease =129 (43.0%), active bleeding=58 (19.3%), malaria =50 (16.7%), visceral leishmaniasis = 25 (8.3%), severe acute malnutrition = 16 (5.30%), snake bite = 11, sepsis = 5, malignancy = 4, hemophilia = 2, thalassemia =2
Akech et al. (2008) (55)	Kenya	Whole blood	NA	65/241 (27%) had symptomatic severe malaria anemia -SSMA. Of these, 64 (98%) received blood transfusion immediately at admission based on the WHO guidelines. Another 45/241 (18.6%) children received blood transfusion later during the course of their admission after meeting the WHO criteria.	Pediatrics	Malaria	The study reported blood transfusion practice for the study cohort (*n* = 241). Medium term survival was ascertained in the 166/213 (78%) patients who returned to follow-up. The overall medium term survival to 196 (92%) children with severe and life threatening malaria who were managed conservatively with respect to blood transfusion in accordance with WHO transfusion guidelines
Akingbola & Bello, 2016 (27)	Nigeria	Whole blood 466 (82.5%)	Blood products Fresh frozen plasma = 5 (0.9%) Packed cell = 5 (0.9%) Cryoprecipitate = 2 (0.4%) whole blood and fresh frozen plasma = 79 (14%) Fresh frozen plasma and packed cell = 2 (0.4%) Whole blood, fresh frozen plasma, and packed cell = 2 (0.3%) whole blood and packed cell = 4 (0.7%)	Transfusion, including blood and blood products = 2.2%	Obstetrics-gynecology	Labor and delivery	Among the 565 obstetric patients, 439 (78.7%) were transfused at delivery and 126 (22.3%) were transfused during pregnancy. Transfusion rate was higher 268 (61.0%) among patients who delivered through CS compared with 165 (37.6%) who had spontaneous vaginal delivery. A higher proportion, 214 (48.7%) of the transfused patients during delivery had emergency cesarean sections, 165 (37.6%) delivered via spontaneous vaginal delivery, 54 (12.3%) delivered via elective CS and only 6 (1.4%) had operative vaginal delivery.
Akinlusi et al. (2018) (34)	Nigeria	Whole blood	NA	20.80%	Obstetrics-gynecology	Labor and delivery	The total frequency of blood units transfused was 6. However, the highest frequency of blood units occurring in 41.3% of patients was 3. Cesarean delivery [Adjusted Odds Ratio [aOR] = 76.14, 95% Confidence Interval [CI] = 1.25–4,622.06, *p* = 0.04], placenta previa [aOR] = 32.57, 95% CI = 2.22–476.26, *p* = 0.01), placental abruption (aOR = 25.35, 95% CI = 3.06–211.02, *p* < 0.001), pre-operative anaemia (aOR = 12.15, 95% CI = 4.02–36.71, *p* < 0.001), prolonged operation time (OR = 10.72 95% CI = 1.37–36.02, *p* < 0.001), previous uterine scar (aOR = 7.02, 95% CI = 1.37–36.02, *p* = 0.02) and hypertensive disorders in pregnancy (aOR = 5.19, 95% CI = 1.84–14.68, *p* < 0.001). Obesity (aOR = 0.24, 95% CI = 0.09–0.61, *p* = 0.002).
Akoko & Joseph, (2015) (83)	Tanzania	Whole blood	NA	105/445 (23.6%)	Specialized care	Urology and general elective surgery	A total of 565 units of blood were cross-matched of which only 27% (153) were transfused. Among 120 patients who underwent elective urology operations, 157 units of blood were cross matched of which 29.9% was transfused to 26% (31/120) of the patients. Among 325 patients who underwent elective general surgical operations, 408 units of blood were cross matched of which 26% (106/408) was transfused to 23% (74/325) of the patients.
Akpa et al. 2022 (33)	Nigeria	Whole blood	NA	620/2034 (30.5%)	General	Sickle cell disease	Prevalence of transfusion transmittable diseases differed or remained stable between cases and controls. The prevalence of hepatitis B virus was similar between controls (6.5%, 95%CI: 5.1 to 8.1%), and cases (5.7%, 95%CI: 4.3 to 7.1%) hepatitis C virus was marginally higher in controls (2.2%, 95%CI: 1.4 to 3.3%) than cases (1.0%, 95%CI: 0.5 to 1.8%); *p* = .048 while the prevalence of Human Immunodeficiency Virus was significantly higher among controls (1.1%, 95%CI: 0.5 to 1.8%) than cases (0.2%, 95%CI: 0.02 to 0.5%); *p* = .022.
Aliyu et al. (2017) (38)	Nigeria	Whole blood	Packed cells, plasma	Whole blood (87.3%) Plasma (0.1%)	Multiple: medicine, surgery, pediatrics, obstetrics and gynecology	Bleeding, anemia, surgery,	The obstetrics and gynecology department (52.3%) had the highest request for blood transfusion, while medicine had the least (9.3%) even though the highest indications for transfusion was anemia (52.2%). Anemia was mostly seen in the pediatrics and medicine departments. Bleeding and surgery was mostly seen in obstetrics and gynecology and surgery departments. Over 90% of requested blood and blood products were dispatched. The average cross-match/transfusion (C/T) ratio was 1.0 while medicine had 1.1, other departments had a C/T ratio of 1.0
Akwiwu et al. (2024) (47)	Nigeria	Whole Blood	NA	67.3%	Oncology	Breast cancer	The majority (62%) of the transfused persons had cytopenia, while those who had anemia alone were 38%. Transfusion of 2 units ranked highest, received by 41%, followed by 1-unit transfusion, received by 35% of the subjects. Three (3) units and 4 units transfusions constituted 8% and 16%, respectively.
Amadi et al. 2023 (29)	Nigeria	Whole blood	Packed red cell	84/1,000 (8.4%) overall obstetric and gynecology 61/828 (7.4%) obstetric and 23/172 (13%) gynecology	Obstetrics-gynecology	Multiple	Hemorrhage was responsible for majority of transfusions in obstetrics, accounting for 40 (65.6%), made up of postpartum hemorrhage 27 (44.3%) and antepartum hemorrhage 13 (21.3%), while chronic compensated anemia alone was responsible for 17 (27.9%), chronic anemia with sepsis 3 (4.9%), and chronic anemia with bleeding 1 (1.6%). In gynecology, chronic compensated anemia was responsible for majority of transfusions 10 (43.5%), while hemorrhage was the reason in 7 (30.4%) and intraoperative bleeding was 6 (21.6%). In obstetric patients, whole blood transfusion occurred in 44 (72.1%) and sedimented blood in 17 (27.9%), while in gynecological patients, whole blood was used in 12 (52.2%) and sedimented blood was used in 11 (47.8%). Blood components used were whole blood 66.7% (56/84) and sedimented blood 33.3% (28/84) only.
Assennato et al. 2018 (81)	Ghana	Whole blood	NA	NA	Pediatrics	Malaria Severe anemia	Nineteen patients (70%) carried male Mc with four (15%) having high levels of Mc (>100 genome equivalent of male cells/million of host cells) com-pared to three controls (37 5%). There was no difference in frequency or quantity of male Mc between pediatric patients with severe malaria and pediatric patients with other causes of severe anemia. TA-Mc was not correlated with patient age, duration of whole blood storage or lymphocyte load transfused. After a median of 7 months post-transfusion, acute malaria did not increase the frequency of TA-Mc.
Asuquo et al. (2025) (45)	Nigeria	NA	NA	70%	Obstetrics and Gynecology	Sickle cell disease	Maternal death was observed among women who received blood transfusions. Blood transfusion during pregnancy were found to be significantly associated (*P* < 0.05) with the type of delivery
Bassey et al. (2024) (46)	Nigeria	Fresh whole blood	Sedimented cells, platelet concentra, fresh frozen plasma	90.63%	Pediatric oncology	Nephroblastoma, rhabdomyosarcoma, non-Hodgkin's lymphoma, acute lymphoblastic leukemia, ovarian tumor, neuroblastoma, retinoblastoma, brain tumor, Hodgkin's lymphoma, osteogenic sarcoma, Ewing's sarcoma, synovial sarcoma, colon carcinoma, nasopharyngeal carcinoma, pancreatoblastoma	Patients less than 5 years of age were the most transfused group. Fresh whole blood was the most transfused component. Leucopenia, was the commonest indication for transfusion in these patients.
Birhan & Asfaw, 2019 (75)	Ethiopia	Blood	NA	NA	Surgical patients	Abdominal, extremity, genitourinary gynecology, head, others, and thoracic.	In the adjusted model, patients showed statistically significant association with preoperative hemoglobin < 11 g/dL (*p* = .000), intraoperative blood loss > 15% (*p* = .000), and neurosurgery (craniotomy) (*p* = .017). Patients with preoperative hemoglobin < 11 g/dL were 7.553 times more likely to have perioperative transfusion than those who had preoperative hemoglobin > 11(AOR = 7.553; CI 2.915–19.576). Patients who had intraoperative blood loss > 15% were 12.830 times more likely to have perioperative transfusion than those who had <15% (AOR = 12.830; CI 5.613–29.323), and among the region of surgery, cranial surgery was 5.868 times more likely to have perioperative transfusion than thoracotomy, abdominal, extremity, genitourinary, gynecologic and other procedures (AOR = 5.868; CI 1.364–25.239).
Bloch et al. (2018) (49)	South Africa	NA	1) Allogeneic red cells 2) Platelets 3) Plasma	NA	Obstetrics-gynecology	Labor and delivery	Cases were more likely to have had no prenatal care (10.2% vs. 2.7%; *p* < 0.0001), be HIV positive (35.4 vs. 28.3%; *p* < 0.0001), having missing CD4 lymphocyte counts, and lower CD4 lymphocyte counts (*p* < 0.0001), have pregnancies with shorter gestational age (*p* < 0.0001), have lower birth weights (*p* < 0.0001), have had obstetric-related hemorrhage either by clinical mention of the diagnosis (67.4% vs. 4.3%; *p* < 0.0001) or based upon estimated blood loss (43.6% vs. 4.5%; *p* < 0.0001), than controls. Gravidity, parity, and rates of caesarean delivery did not differ significantly from those of controls. Two deaths occurred in the transfused group vs. one death in the non-transfused group.
Bolton et al. 2021 (51)	South Africa	NA	Red blood cells. # of RBC transfusion event = 23,56,441 # of RBC units issued between 2014 and 2018 = 38,99,389	Transfusion events in the public secto*r* = 65.9% Transfusion events in the private secto*r* = 34.1%	NA	NA	Recipients in the public sector were younger (median [interquartile range (IQR)] 33 (22–49) years) than in the private sector [median (IQR) 54 (37–68) years], and predominantly female (66.2% v. 53.4% in the private sector). There was a 1:2 ratio in the transfusion events for males v. females in the public sector, compared with a ∼1:1 ratio in the private sector. Public sector recipients were predominantly younger males aged 0–4 and females aged 20–39 years old. In the private sector, transfusion events increased with age and occurred mostly in men in the 55–59 year age group and women older than 80 years.
Bugge et al. 2012	Malawi	104 units of whole blood	NA	NA	Pediatrics, Obstetrics-gynecology, and men's health	Malarial anemia, pregnancy-related anemia, surgical cases, HIV-related anemia, Other anemia.	more than half of the Transfusions were given to patients diagnosed with malaria, and 93% (55 ⁄ 59) of these were pediatric patients. Sixty-four percent (66 ⁄ 104) of all blood units were given at the pediatric unit, and 29% (30 ⁄ 104) were given to adult women; of which 60% (18 ⁄ 30) were given for pregnancy-related reason.
Chansa et al. 2014 (77)	Zambia	Intraosseous access (IO) blood transfusion	NA	NA	Pediatrics	Anemia	At admission, the Haemoglobin level was 3.6 g/dL, the white blood count was 23.3 109/L and the platelet count was 178 · 109/L. At the return visit one week later, the haemoglobin level was 6.0 g/dL. Threemonths later, the haemoglobin level was 9.4 g/dL and the child was doing well.
Checkley et al., 2019 (86)	Uganda	Whole Blood	NA	Children: 33.2%; Female 55.7%	Not mentioned	Not mentioned	373 blood transfusions were recorded at the health facility over 46 days. 55.8% of blood units were allocated to female patients and 33.2% were allocated to children under five years old. Key themes regarding community members knowledge and awareness of blood donation include: 1) attitudes towards blood, 2) motivations of blood donation, 3) deterrents of blood donation, 4) blood donation safety, 5) lack of information.
Chiabi et al. (2024)	Cameroon	Whole blood	Packed red blood cells	4.8%	Pediatric	Various health conditions	The majority of transfused children, 95 (30.8%), were aged 1–3 years. A total of 298 patients (96.8%) received whole blood transfusions, while 10 patients (3.2%) received packed red blood cell transfusions. Most patients, 175 (56.8%), received one transfusion, followed by 91 (29.5%) receiving two transfusions. The mean number of transfusions was 1.59 ± 0.792. Post-transfusion reactions developed: Urticaria 27 (8.8%) was relatively common, followed by febrile reactions 15 (4.9%).
Dhabangi et al. 2019 (76)	Uganda	Blood	Blood products (not described)	NA	Pediatrics	Severe anemia	Participants noted that: 1) blood transfusion is a life-saving treatment for severe anemia. 2) Fears of perceived risk associated (e.g., HIV, blood incompatibility, negative effects on cognitive abilities and behavior, and persistent risk of future transfusion with children receiving blood transfusions. 3) challenges in accessing blood for transfusion. These include distance to health facilities, weak triage systems, costs, unscrupulous and corrupt behaviors of some healthcare workers, inaccessible hospitals, and scarcity. 4) fears and consequences of blood transfusion, including community misperception. 5) Peri-transfusion and post-transfusion care, beliefs and practices.
Diaku-Akinwumi et al. (2016)	Nigeria	Whole blood	True packed red blood cell Washed red cells leukocyte-depleted cells	NA	Adult and Pediatrics	Sickle cell disease	Very few hospitals provide blood transfusion through centrally coordinated blood banking regulatory systems, with most administering transfusions at their discretion. Most centers also depend on commercial or family donors. Hence, it is not surprising that they often experience blood scarcity. No center offering CTT screened for syphilis. The efficacy of CTT transfusion is sub-optimal. Only two hospitals employ top-up transfusion while others offer exchange blood transfusion manually via large peripheral veins, signaling a suboptimal CTT practice. Only ABO and Rh D cross-matching were done, and no screening of other red cell antigens or alloantibodies was reported. Most hospitals did not produce leukocyte-depleted packed red cells, which raises concerns for safety from diverse transfusion reactions. A quarter of the hospitals could monitor iron overload, but only using serum ferritin. Access to iron chelators was limited and expensive. Seventeen (55%) tertiary hospitals offered CTT by top-up or manual exchange transfusion; previous stroke was the most common indication.
Drammeh et al. (2018) (67)	Tanzania	Whole blood	Fresh frozen plasma, packed red blood cells, and platelets.	14,706 blood transfusion requests for 14,698 patients. 16,728 components issued to patients 21,409 requested components Whole blood = 17,163 (80.2%)	General	Adult and children	Of the components requested, 2,453 (11.5%) were for adults packed red blood cell, 1,165 (5.4%) for pediatric packed red blood cell, 321 (1.5%) for frozen fresh plasma, and 307 (1.4%) for platelets. 767 (5.2%) of the 14,698 patients who requested for blood products died during the first 7 days of hospitalization.
Efobi et al. (2021) (21)	Nigeria	NA	NA	NA	NA	NA	30.4% did not keep a comprehensive transfusion record, 66.7% screened all patients for transfusion-transmissible infections at first contact, 79.7% did not determine red cell antibody status at first transfusion, 94.2% did not use leukodepleted red cells, and 75.4% reported poor practice of multiple blood transfusions. Those with a blood transfusion policy in their centers appeared to have better knowledge of safe blood transfusion than those without, *P* = 0.008.
Eyelade et al. (2015) (26)	Nigeria	Whole blood	NA	64/642 (9.1%)	Obstetrics-gynecology	Hemorrhage	The likelihood of blood transfusion [Odd ratio (OR)] was highest when estimated blood loss of at least 1,000 mL was used as control regression model 1[OR = 43.07; 95% Confidence Interval (C.I.) = 17.22–107.76]. The likelihood of blood transfusion was higher in participants with preoperative PCV < 26% when this was used as control in regression model 2 (OR = 33.8, 95% C.I. = 11.8–97.0). While blood is more likely to be transfused in women who had antepartum hemorrhage, the percentage of women who received blood transfusion increased with the estimated blood loss in milliL (mL).
Fenta et al. (2024) (63)	Ethiopia	NA	NA	NA	NA	NA	Facilities offering blood transfusion services had a mean readiness score of 4.5 (out of 7) based on the WHO Service Availability and Readiness Assessment guide, with only 5% having all items. Most facilities performed blood typing, but less than one-third conducted cross-match testing, and over half lacked guidelines and trained staff. Service readiness varied significantly across regions. Increased availability of medical equipment corresponded to a 49% increase in readiness scores [Coef.: 0.49; 95% CI: (0.19, 0.79)].
George et al. 2022 (71)	Uganda and Malawi	Whole blood	Red cell concentrates	NA	Pediatrics	Severe anemia	2,692 (84%) children received one transfusion, 349 (11%) received two transfusions, and 147 (5%) received three or more transfusions during initial admission. Whole blood was the first pack provided for 1,632 (41%) of 3,992 transfusions, of which 1,101 (67%) of 1,632 were adult-size packs and 531 (33%) were from transfer packs (pedipacks). 844 (36%) of 2,360 red cell concentrate transfusions were packed cells whereas 1,516 (64%) were settled cells.
Gyedu et al. (2021) (74)	Ghana	Whole blood	NA	458/1,116 (41%)	Accident and Emergency Center	Injuries	More than half (60%) of the patients who received transfusion had sustained injuries due to road traffic crashes compared to those who were not transfused (54%). More burn injury patients were transfused (2% vs. 0.9%). Additionally, transfused patients had a higher proportion of multiple serious injuries (24% vs. 16%, *p* < 0.001) and had worse Kampala Trauma Score II (44% moderate or severe injuries vs. 32%, *p* = 0.002), respectively.
Ijah et al. (2024) (40)	Nigeria	NA	NA	NA	General surgery	Trauma, infection, metabolic, neoplastic, intestinal obstruction	The Transfusion Index (TI) was 1.13 for 2022 and 1.66 for 2023. Intestinal obstruction had the highest number of intraoperative blood transfusions (*n* = 32). Exploratory laparotomies performed for non-trauma cases had the highest number of blood transfusions (*n* = 66), followed by mastectomies (*n* = 24), and emergency exploratory laparotomies done for trauma (*n* = 12)
Jacobs et al. (2023) (44)	Rwanda, Senegal, Sudan, Tanzania, The Gambia, Uganda, Zambia, Zimbabwe, Botswana, Burundi, Cameroon, Cote d'Ivore, Egypt, Eswatini, Ethiopia, Ghana, Kenya, Lesotho, Liberia, Malawi, Mozambique, Nigeria	whole blood	Red blood cells (RBCs), platelets, plasma, and cryoprecipitate	NA	NA	NA	Only four countries had a responding institution that provides all types of products (Kenya, Malawi, Nigeria, and Uganda). Whole blood is the only available blood product at institutions in four countries (Burundi, Cameroon, Sudan, and The Gambia), and only RBCs are available at institutions in Lesotho and Zimbabwe. Cryoprecipitate is the least common blood product provided: only 12.4% (10 of 81) of institutions supply it. Among all 81 institutions, regardless of whether blood is collected onsite or obtained from an outside supplier, 66 (81.5%) test for at least one infectious agent, the most common of which are human immunodeficiency virus (HIV) (81.5%, 66 of 81) and hepatitis B virus (HBV) (81.5%, 66 of 81).
Jatau et al. (2023)	Nigeria	Whole blood	NA	NA	Multiple	Multiple	3,844 (44.97%) blood requests made were never utilized, while the highest number of units transfused was 14 units. Units of blood requested for transfusion ranged from 1 unit to 10 units per request. However 2 units accounted for 4,668 (54.56%) of all requests. The highest number of requests 2,000 (23.4%) was received from the department of Obstetrics and Gynecology while the least request was from dermatology.
Kintieti et al. (2026)	Democratic Republic of Congo	NA	NA	20.2%	Orthopedic care	Anesthesia	Factors independently associated with transfusion were hemoglobin ≤10 g/dL, ASA III-IV status, intraoperative incidents, major surgery, operative time ≥2 h, conjunctival pallor and frailty/dependence. On the other hand, alcohol consumption appeared to be a protective factor.
Keating et al. (2021) (13)	Malawi	Whole blood	NA	989/1,761 (56.2%)	Pediatrics	Severe anemia and malaria	The overall in-hospital mortality rate for this cohort was 7.2%. The overall In-hospital mortality rate for patients who received a blood transfusion was 7.8% (60 of 772 patients) and for those who did not receive a blood transfusion was 6.7% (66 of 989 patients). The mortality rate in patients who received a single transfusion was 7.4% (63 of 852 patients) and in those who received several transfusions it was 2.2% (3 of 137 patients).
Kiguli et al. (2015) (57)	Kenya	Whole blood Among severely transfused anemic patients 1,459/1,767 (83%).	Packed red blood cells At only one hospital 308/1,767 (17%)	1,387/3,082 (45%)	Pediatrics	Anemia	Among surviving severely anemic children, 94% were transfused by 8 h, 96% by 24 h, and 99% by 48 h of admission. The proportion of moderately anemic children who were transfused increased rapidly during the first 2 h of admission, and again between 8 and 10 h, following repeat Hb measurement at 8 h. Among 843 children who were mildly anemic on admission, 4% were transfused by 8 h, and 8% by 24 h. Hb at baseline (Hazard ratio [HR] 0.84 (0.74–0.95), severe pallor [HR 3.18 (2.42–4.19)], jaundice [ HR 1.27 (0.97–1.68)], capillary refill time ≥3 s [HR 1.59 (1.24–2.03)], lactate [HR 1.08 (1.05–1.10)], and chest indrawing [HR 1.30 (1.01–1.69)] were independent predictors of transfusion.
Linstrom et al. (2024)	South Africa	Whole blood	Platelet, plasma units, packed red blood cells	Prevalence by age: <19 years (35.4%), 19–24 (24.9%), 25–29 (25.7%), 30–34 (23.8%), 35–39 (23.0%), >40 (26.9%)	Obstetrics and Gynecology	Anemia, leukocytosis, thrombocytopenia	Hb <10 g/dL (OR = 6.41; 95% CI 5.46–7.71), was the strongest predictor of transfusion. During this study period, there were 33 reported maternal deaths. The probability for mortality in a patient exposed to blood transfusion was increased in comparison to patients without a history of blood transfusion (OR = 3.60; 95% CI 1.75–7.47). Patients presenting with an Hb below 10 g/dL were four times more likely to die compared to those with Hb above 10 g/dL (OR = 4.15; 95% CI 2.03–8.49).
Mafirakureva et al. (2015) (72)	Zimbabwe	whole blood	Patients who received red blood transfusion 1,642/1793 (91.6%). Most of the transfused components were red blood cells (*n* = 3,660; 86.1%) followed by fresh-frozen plasma (*n* = 444; 10.4%), platelets (*n* = 93; 2.2%), and cryoprecipitate (*n* = 32; 0.8%).	Units transfused 4,249 (15.0%) of blood components. (*n* = 20; 0.5%).	General	NA	Of all the blood components transfused, the majority were transfused to women. Transfusion recipients in the reproductive age group (15–49 years) received 2,560 (69.9%) units of red blood cells, 372 (83.8%) units of fresh-frozen plasma, 62 (66.7%) units of platelets, 9 (45.0%) units of whole blood and 30 (93.8%) units of cryoprecipitate. Paediatric patients below the age of 5 years received 155 (4.2%) units of red blood cells, 13 (2.9%) units of fresh-frozen plasma; 15 (16.1%) units of platelets and 6 (30.0%) units of whole blood. Transfusion recipients aged 65 years or older received the following units: red blood cells (*n* = 394; 10.8%), fresh-frozen plasma (*n* = 16; 3.6%) and whole blood (*n* = 2; 10.0%).
Mafirakureva et al. 2016 (88)	Zimbabwe	Useful whole blood collected = 67,422	Number of blood components prepared from whole blood was 69,242. 1) Red blood cell 2) Fresh-frozen plasma 3) platelets	62,303/69,242 (90.0%) of blood units were distributed.	NA	NA	The overall unit production cost of RBCs in Zimbabwe for 2013 was US$130.94, constituting 13.7% of the country's GDP per capita.
Mandar et al. 2022 (68)	Eastern Sudan	Whole blood	NA	44 (8.2%)	Obstetrics-gynecology	Labor and delivery	A significant association between emergency CD (AOR=2.57, 95% CI = 1.25‒5.28) and antepartum hemorrhage (AOR=44.70, 95% CI = 11.18‒178.76) with post-CD transfusion. Preoperative hemoglobin (AOR=0.48, 95% CI = 0.36‒0.64) and rural residence (AOR=0.45, 95% CI = 0.22‒0.93) were associated with a lower risk of postpartum blood transfusion.
Morris et al. 2019 (50)	South Africa	Whole blood	NA	NA	Multiple	Multiple emergency care	329 units of emergency blood were transfused to 210 patients. 141 transfusion episodes with 186 transfusion units (1:3) units/episode ratio occurred at the secondary hospital, and 69 with 143 units (2,0) units/episode ratio occurred in the tertiary hospital. Trauma (26% episodes and 31% unites and 65% episodes and 67% units, at the secondary hospitals and tertiary hospitals, respectively). Surgical (27% episodes and 26% unites and 13% episodes and 10% units, at the secondary hospitals and tertiary hospitals, respectively). Gynecology (21% episodes and 21% unites and 4% episodes and 3% units, at the secondary hospitals and tertiary hospitals, respectively). Medical (21% episodes and 17% unites and 1% episodes and 61% units, at the secondary hospitals and tertiary hospitals, respectively). Obstetrics (9% episodes and 10% unites tertiary hospitals.) Others (7% episodes and 8% unites tertiary hospitals.) unaccounted for
Mumo et al., 2023 (56)	Kenya	Whole blood	NA	NA	Not mentioned	Not mentioned	The average travel time from any population location to transfusion facility was 33 min. Vulnerable populations in some counties had over 1-hr travel time to the transfusion facility. 33.5%, 27.8%, and 4.9% of marginalized patients across three counties traveled for over 1hr to the transfusion facility.
Musa et al. (2014) (36)	Nigeria	Whole blood	NA	NA	Multiple	Multiple	1,703 units were requested for 986 patients during the study period. About 94.42% (1,608) of the requested units were crossmatched while only 41.73% (671) and 34.51% (555) of the crossmatched units were issued out and transfused, respectively. This gave a CTR of 2.90 for the hospital. Obgyn had the highest request 738 (43.34%) while the medicine unit had the lowest request 107 (6.28%). Similarly, the obgyn unit had the highest CTR of 3.40 while Medicine and Pediatrics units had a CTR of about 2 or less.
Nabwera et al. (2016) (54)	Kenya	Whole blood	NA	NA	Pediatrics	Anemia	Ninety percent (1,505/1,663) were presumed transfused. Mediantime from laboratory receipt of request to collection of blood was 3.6 h (IQR, 1.4–12.8 h). Case notes of 590 children were reviewed and median pretransfusion hemoglobin level was 6.0 g/dL (IQR, 4.2–9.1 g/dL). 67% (396/590) of these children received a blood transfusion.
Natukunda et al. (2010) (84)	Uganda	Whole blood	Red blood cells	75.6%	Multiple: Pediatrics, medical, obstetrics-gynecology, accident and emergency, surgical	Multiple	Blood was ordered and cross-matched for 1,730 patients but 1,674/1,730 (96.8%) were transfused. The three most frequent indications for blood transfusions were malaria (38.8%), bleeding (27.1%), and other infections (16.1%). More children 672/556 (82.7%) than adults were mainly admitted for malaria. Obstetric hemorrhage accounted for 55·7% of all bleeding patients who received transfusions and for 75·4% of all recipients at the OBGY ward. 3,673 units of whole blood and RBCs were ordered and out of these, 2,777 units (75·6%) were transfused. 58·4% of all recipients were given whole blood transfusions. Approximately 97·0% of the Pediatric ward recipients were transfused with packed red blood cells (RBCs). There were 5 additional units of random donor platelet transfusions in two recipients (0·4%)
Nisingizwe et al. 2022 (80)	Rwanda	NA	Blood products	NA	NA	Emergency care	Between March 17, 2017, and Dec 31, 2019, 12,733 blood product orders were delivered by drones. Overall, the mean delivery time by drone was 49·6 min (95% CI 49·1–50·2), including order preparation and packaging time. By contrast, road delivery took a driver a median time of 120 min (IQR 120–180) to deliver a blood request order back to the health facility. When using Google Maps, the estimated driving times, the median road delivery time would have been 139 min (IQR 87–175), whereas drone delivery had a median time of 41 min (IQR 33–49) excluding order preparation. Drone delivery was 79 min (*p* < 0·0001) faster using estimated driving times and 98 min faster (*p* < 0·0001) based on Google Maps estimates.
Njolomole et al. 2022 (79)	Malawi	Blood	Blood products	NA	Obstetrics-gynecology	Postpartum hemorrhage	1) Availability of blood products, including the lack of adequate blood supply and cultural belief were barriers against timely blood transfusions for patients. 2)Transport of blood products or transfer of patients to target sites. These issues were related to transportation and availability of vehicles and distance between the lab and the maternity unit for timely transportation of patients blood from the blood bank to the facilities. 3) District hospital resources and procedures. Related to logistical and administrative burdens. Lack of funding to purchase equipment and to employ personnel. 4) Clinical protocols and practice related to risk assessment of patients to either avoid over burdening or delaying requests. 5) Communication between health cadres involving the miscommunication between health cadres involved in the delivery process.
Nwafor et al. 2018 (32)	Nigeria	NA	Fresh frozen plasma Platelet concentrate Packed cells	NA	Surgical care	Open heart surgery	Transfusion requirements averaged 1.9 units of fresh frozen plasma, 0.36 units of platelet concentrate, and 1.68 units of packed cells per procedure. The least common surgical procedure was common atrium repair (*n* = 1, 0.01%).
Okoroiwu & Okafor (2018) (35)	Nigeria	Whole blood	Packed cells and plasma	NA	Multiple	Multiple	473 units were requested consisting of 1,770 whole blood, 468 packed cells and 235 plasma. About 94.46% (2,336) of the cross matched requests were issued consisting of 1,636 (70.04%) whole blood, 467 packed cells (19.99%) and 233 (9.97%) plasma resulting in a cross match to transfusion (C/T) ratio of 1.06.
Olupot-Olupot et al. 2016	Uganda	Whole blood	NA	282/318 (88.7%) 94 of 282 (33.3%) and 9 of 282 (3.4%) received two or three transfusions, respectively.	Pediatrics	Fever	Overall, 282 of 318 cases (88.7%) received a transfusion compared with 700 of 1,552 controls (45.1%) (*P* < .0001). A higher proportion of cases than controls received a second and third transfusion 94/282 [33.3%] vs. 107/700 [15.3%] and 9/282 [3.4%] vs. 0/700 [1.4%], respectively; *P* < .0001.
Opoka et al. (2018) (69)	Uganda	Whole blood	NA	551/574 (95.9%) diagnosed with severe anemia. accounted for 98.4% of all transfusions in children 0–5 years in the two hospitals	Pediatrics	Severe Anemia	Of the blood transfusions given to children assigned a diagnosis of SA (44.5%) were given appropriately as per guidelines (pre-transfusion Hb ≤ 6 g/dL), while 306 (55.5%) were given inappropriately, [pre-transfusion Hb not done, *n* = 216, or transfusion not indicated (Hb > 6.0 g/dl), *n* = 90]. Of the SA children not transfused, 22 were referred to other centres because of shortage of blood while one child died before receiving a blood transfusion.
Oreh et al. (2022) (39)	Nigeria	Whole blood	Red cells, platelets, fresh frozen plasma	NA	Multiple: Obstetrics-gynecology, Pediatrics, General Surgery, Accident and Emergency, Internal Medicine, other departments).	NA	The mean (± standard deviation) amount of blood transfused in January to July 2019 before COVID-19 was 14,344 ± 790 units per month, while the mean (± standard deviation) amount of blood transfused in January to July 2020 was 11,235 ± 1,985 units per month showing 21.7% decrease in blood transfusion *P* = 0.021. This study clearly reveal that the COVID-19 pandemic negatively affected the numbers. of blood donations and blood transfusions—obstetrics and gynecology, a 53.5% decline was observed in the transfusion of platelets over the study period (*p* = 0.041). Internal medicine departments reported declines of up to 48.1% in red cell transfusions (*p* = 0.014). The highest declines in blood transfusions by hospital department and blood product were observed in surgery department transfusions of fresh frozen plasma (80.1%) (*p* = 0.012) and accident and emergency department transfusions of platelets (78.3%) (*p* = 0.005). The least decline was observed in surgery department. platelet transfusions (2.7%).
Patidar et al. 2022 (30)	Multiple countries: Democratic Republic of Congo, Ethiopia, Kenya, Morocco, Nigeria, and Rwanda	Whole blood	Blood components Leuko-reduced blood components Fresh frozen plasma Platelet concentrates plateletpheresis procedures	NA	NA	NA	Use of whole blood in Zimbabwe was 10%, 11–20% in Rwanda and Democratic republic of Congo, 50% in Ethiopia, and up to 80% in Nigeria.
Pitman et al. 2015 (61)	Namibia	NA	Blood components, *n* = 3 (red blood cells, platelets, and plasma). Blood components units analyzed, *n* = 91,207. Red blood cell requested and issued, *n* = 78,660 Fresh frozen plasma requested and issued, *n* = 9,751 Platelets issued, *n* = 2,978	NA	Multiple	Multiple	The indications for blood component transfusion varied with red blood cell being the most requested and issued. 10% of all platelet units were ordered for children with malignant neoplasms highlighting changing diagnostic and clinical practices and capacity in Namibia. Diagnoses in the malignant neoplasms (C00-C97) category accounted for 38.1% of the 2,978 platelet units issued during the study period. Nearly one-quarter of these units (2,251 units; 23.1%) were issued for diagnoses in the gastrointestinal (K20-K93) diagnostic category. 38.9% (*n* = 30 616 units) were issued for diagnoses in the diseases of the blood and blood-forming organs.
Reggiani et al. 2019	Mozambique	Whole blood	Red blood cell concentrates Platelet concentrates Fresh frozen plasma Platelet-rich plasma Modified plasma Two hemocomponents	NA	Pediatrics and neonatology	Severe anemia and malaria	Indications for transfusion were sepsis in the Neonatology (41% of neonatal requests) and malaria in the Pediatric Emergency units (39% of paediatric requests). Of the 1,594 fully completed forms, 1,158 adhered to WHO indications. 22% of the red blood cell concentrate' recipients > 2 months of age had an Hb level >6 g/dL, had no indication for transfusion according to WHO guidelines. Adherence to WHO indications was significantly related to age (*P* = .0153), appearing the lowest in infants. About 3% of the requests were not attended to, and there was no statistically significant difference in this percentage between children with and without WHO indication for transfusion (*P* = .38).
Salami et al. (2022)	Nigeria	Whole blood	NA	95/693, 13.7%	Obstetrics-gynecology	Labor and delivery	Concerning caesarean delivery type, 7.8% and 5.9% of those who had elective and emergency lower segment cesarean section were transfused respectively. Ninety-five women (25.7%) of patients that had cesarean sections were transfused. Chi square test revealed that preoperative packed cell volume, age and caesarean delivery type were significantly associated with blood transfusion status (*p* < 0.05).
Sawadogo et al. (2020) (59)	Burkina Faso	Whole blood	NA	98% (343/350)	Pediatrics	malarial anemia	352 blood orderings were issued for the 315 children. The median actual time to transfusion was 65 min (IQR: 45–100) and was associated to the presence of non-tolerated anemia (*p* = 0.005). When considering the different sequences of this actual time to transfusion, only in-service blood conservation time was significantly associated to the degree of emergency and the presence of non-tolerated anemia. With a median of 73.8 min (IQR: 47.5–110), transfusion duration was associated to the degree of emergency.
Shari et al. 2017 (66)	Tanzania	Whole blood	NA	23.20%	Pediatrics	Anemia	125 (48.6%) children had WHO-defined indications for blood transfusion at the time of ED presentation. Of these children, 45 (36%) had Hb <4 g/dL, 76 (60.8%) had Hb between 4 g/dL and 7 g/dL with shock, and 4 (3.2%) had Hb > 4 g/dL with continuous bleeding. 85 (68%) of the children with WHO-defined indications for blood transfusion, and ordered blood in 58 (68%).
Tadeu & Geelhoed, (2016) (73)	Mozambique	NA	NA	NA	Multiple services requiring laboratory services.	NA	Physicians often needed to transfer women with childbirth complications to usally distant facilities with readily available blood transfusion services. This has led to maternal death because of difficulties in arranging timely transfusions. Issues with increased blood transfusion capacity, CD4, and supply of reagents because of stock-outs.
Tewabe et al. (2022) (82)	Ethiopia	Whole blood	Plasma, platelet, packed red blood cell	NA	Multiple	Multiple	1,540 blood units were requested for 616 study subjects. All 1,540 blood units were cross-matched and 1,498 blood units were transfused, with a mean of unit 2.43 CT ratio. 110 (17.9%) of the patients were given four units of blood, 196 (31.8%) were given three units of blood, 160 (26.0%) were given two units of blood and the rest 150 (24.4%) were given one unit of blood.
Thomas et al, 2017 (7)	Kenya	Whole blood	Packed red cell	2,875 blood transfusion was ordered. Receipt of blood transfusion was among 2,352 patients. Eight of 10 (82%, 2,352/2,875) of all the orders for transfusion at admission result in blood administration, with 75% (1,760/2,352) of these children being transfused on the same day.	Pediatrics	Malaria, anemia, severe anemia, sickle cell disease, malnutrition.	The number of blood transfusions prescribed within hospitals during the period ranged from 14 to 1,032. Blood transfusion prescriptions that were actually administered per hospital ranged between 71% and 100%. Seven percent (3,486/53,174) of admissions in the 10 hospitals died, and 13% (381/2,875) of these deaths occurred in children for whom a transfusion was ordered. Among the patients with blood transfusion prescribed, the group which was not transfused witnessed the highest mortality of 20% (105/523) compared with 12% (276/2,352) among those transfused.
Tort et al. (2015) (60)	Senegal and Mali	Whole blood	Red blood cells	(907/3,278) 27.7% of women received a transfusion.	Obstetrics-gynecology	Postpartum hemorrhage	After adjustment for women's characteristics, aspects of pregnancy and delivery, the risk of death for women with PPH was 2.17 higher in women with transfusion. Adjusted odds ratio [(2.17) 95% CI: 1.53–3.09] and case fatality ratio 9.0%
Tsima et al. (2016) (62)	Botswana	Whole blood and red cell concentrate.	Plasma and platelet	11%	Obstetrics-gynecology	Labor and delivery	The number of patients who received one or more units of whole blood or red cell concentrate (RCC), plasma from fresh frozen plasma (FFP) and platelets were 59 (9·5%), 8 (1·3%) and 4 (0·7%), respectively. A total of 15/55 (27·3%) of the patients who received blood transfusions received only 1 U of blood. Patients who received two units of blood transfusions comprised the largest share of transfused patients (31/55; 56.4%). Blood and blood components were used infrequently in the management of post-abortion complications. There was a moderate positive correlation between admission hemoglobin level and time to transfusion, The number of blood units given increased with decreasing admission hemoglobin level.
Uche et al. (2025) (43)	Nigeria	Whole blood	Packed cells, platelets, fresh frozen plasma, cryoprecipitate	NA	Pediatrics, internal medicine, surgery, obstetrics and gynecology, and accident and emergency	NA	None of the facilities had a multispecialty transfusion service, and 100% lacked a transfusion committee. 15.4% had an existing quality policy on blood issues, and none had a quality officer. Blood components, like packed cells, platelets, and cryoprecipitate, were unavailable in all the institutions. Equipment, such as standard blood banks (30%), domestic freezers (23%), and blood agitators (7.5%), were inadequately available, and most cross-matching was conducted using saline at room temperature (85%).
Umar et al. (2024) (42)	Nigeria	Whole blood	Packed cell/red cell concentrate, platelet concentrate, fresh frozen plasma, cyoprecipitate	NA	NA	NA	The majority (68%) of the tertiary hospitals lack facilities for blood component preparation and only 18% and 32% provide cryoprecipitate and platelet concentrate, respectively. Whole blood was most commonly requested (57.04%). The average time to process routine, emergency and uncross-matched requests were a mean of 109.58 ± 79.76 min (range 45.00–360.00), 41.62 ± 25.23 (10.00–240.00) and 11.09 ± 4.92 (2.00–20.00), respectively.
Weeber et al. (2018) (52)	South Africa	NA	NA	NA	Emergency	Injuries	The vast majority of patients were estimated to not have injuries severe enough to mandate transfusion (*n* = 275, 93.5%; ISS median:5, IQR:3–9). Of the 19 patients that were estimated to require transfusion(*n* = 19, ISS median of 14 (IQR:12–19, 15 (57.9%) would have needed only one unit. There were four patients that were estimated to require more than one unit. Of these, two patients were estimated to have suffered massive blood loss (more than 50% circulation blood volume).
Wentzel et al. (2019) (53)	South Africa	NA	Overall, 56% (98/176) were treated with fresh frozen plasma. Of these, 54% (44/81) were South African.	NA	Specialized care	Angioedema	Time from arrival at the emergency room to the start of FFP infusion was a median (IQR) of 2 h (0.5–3); with longer delay in South Africa than Iran [South Africa: 3 (2–4.9) hours vs. Iran: 1 (0.3–2.5) hours. South African episodes, 43.1% (18/44) patients arrived after 12 h from symptom onset.

#### Prevalence of transfusion

As seen in Table 2, the prevalence of transfusion differed across countries, types of care, and indications. However, transfusion prevalence in pediatrics superseded other healthcare domains. Anemia-related illnesses accounted for the highest prevalence of pediatric transfusions at 98.4% in Uganda (69). Pediatric transfusions for malarial anemia (343/350; 98%) was consistently high in Burkina Faso (59) and Uganda (65), and the lowest prevalence of 4.8% among pediatric patients with multiple indications was reported in Cameroon (87). In women's health, gynecological health services had the lowest transfusion prevalence of 2.2% (27), and postpartum hemorrhage (PPH) had the highest at 27.7% (60) in studies of Senegalese and Malian mothers.

### Types of care and indications for blood transfusion in SSA

Pediatrics care accounted for almost half of the services needing blood and blood component transfusion (*n* = 27/65; 41%) (7, 13, 31, 36, 38, 39, 43, 46, 54, 55, 57, 59, 61, 65–67, 69–72, 76–78, 81, 84–87). The indications varied, with most being anemia-related: severe anemia, malarial anemia, and anemia in sickle cell disease (*n* = 15/27; 55%). Other pediatric indications included severe cancer, acute malnutrition, septicemia, and severe pneumonia. Women's healthcare was the next most reported type of care. Twenty-two studies (*n* = 22/65, 33%) (26–29, 34, 36–39, 43, 45, 48–50, 58, 60, 62, 68, 72, 78, 79, 84) accounted for obstetric and gynecological care. The most common indication for transfusion among women was hemorrhage in the postpartum and antepartum period (*n* = 6/22; 27%). Next was labor and delivery, comprising transfusion received during vaginal or cesarean birth and post-abortion care (*n* = 5/22; 22%). Other types of care reported were related to general medicine and care, specialty care, such as surgery—trauma, abdominal, extremities, urology, neoplasms, and injuries associated with emergency unit care (Table 2).

## Barriers to blood and blood component transfusion using the WHO health systems framework

### Service delivery

In our study, out of thirty-three (*n* = 33) articles that reported systemic barriers, twenty-nine (*n* = 29/33, 87%) (7, 27, 29–31, 36, 37, 41–44, 52–54, 56, 57, 60, 63, 66, 67, 69, 70, 73, 74, 76, 79, 83, 84, 86) reported several service delivery barriers that impede blood and blood product use across SSA, Table 3. These were low transfusion rates, inappropriate transfusion practices, delayed transfusion, and untimely referrals. Shari and colleagues (66 noted that despite the high prevalence of moderate-to-severe anemia in children under five, transfusion rates were as low as 23.2% in emergency departments. In Nigeria, most of the women presenting for labor and delivery had only one unit of blood transfused despite the need for more units based on estimated blood loss at delivery and markedly low hematocrit or packed cell volume (PCV) values 27. Even where high transfusion rates were reported, Amadi et al. (2023) (29) noted the possibility of inappropriate transfusion practices as a quarter of the transfused patients had pretransfusion PCV of ≥25% (transfusion threshold ≤20%). Similarly, at least 16% of children with anemia were inappropriately transfused despite having pre-transfusion hemoglobin concentration (Hb) levels > 6 g/dL (transfusion threshold ≤ 4 g/dL) (69). Tort and colleagues (60), noted that untimely referrals and transportation challenges increased the odds of poor health outcomes and led to case-fatality ratios of up to 50% in women with PPH. Being transferred to another hospital was significantly associated with maternal mortality (adjusted odds ratio 13.35). Uche and colleagues reported that none of the 13 institutions represented in their study provided multispecialty services and used substandard blood transfusion protocols (43). The time interval between when a patient came to the hospital and when they were transfused was also a barrier. In Kenya, Nabwera and colleagues (54) found a 3.6- and 5.4-hour median request time from laboratory receipt to blood collection for children with severe anemia. This delay was significantly shorter in children with severe anemia compared to those without. Similar findings were reported in Nigeria, where the average process time for routine, emergency, and uncross-matched requests was a mean of 109.58 ± 79.76 min (range 45.00–360.00), 41.62 ± 25.23 (10.00–240.00), and 11.09 ± 4.92 (2.00–20.00), respectively (42).

**Table 3:** Barriers of blood and blood product access using the WHO health system building blocks.

Author/Year	Title	Country	Service Delivery	Health Workforce	Health Information Systems	Access to Essential Medicines	Financing	Leadership/Governance
Ahmed et al. 2019 (70)	Severe childhood anemia and emergency blood transfusion in Gadarif Hospital, eastern Sudan.	Eastern Sudan	**+**					
Akoko & Joseph 2015 (83)	Blood utilization in elective surgery in a tertiary hospital in Dar es Salaam, Tanzania.	Tanzania	**+**	**+**	**+**	**+**		**+**
Akingbola & Bello 2016 (27)	Obstetric emergencies and transfusion needs in a Nigerian hospital.	Nigeria	**+**			**+**		
Amadi et al. 2023 (29)	Evaluation of Blood Transfusion Practice in Obstetrics and Gynecology at a Tertiary Hospital in Port Harcourt, Nigeria.	Nigeria	**+**					
Bugge et al. 2012	A study of blood transfusion services at a district hospital in Malawi.	Malawi				**+**		
Checkley et al. 2019 (86)	Assessment of blood donation and transfusion in Eastern Uganda: A mixed-methods study.	Uganda	**+**			**+**	**+**	**+**
Diaku-Akinwumi et al. 2016	Blood transfusion services for patients with sickle cell disease in Nigeria.	Nigeria	**+**		**+**	**+**	**+**	**+**
Dhabangi et al. 2019 (76)	Caregivers and community perceptions of blood transfusion for children with severe anemia in Uganda.	Uganda	**+**		**+**	**+**	**+**	**+**
Drammeh et al. 2018 (67)	Estimating Tanzania's national met and unmet blood demand from a survey of a representative sample of hospitals.	Tanzania	**+**			**+**		**+**
Efobi et al. (2021) (21)	Snapshot on physicians' view on safe blood transfusion in multiply transfused patients in Nigeria	Nigeria	+			+		
Fenta et al. (2024) (63)	Blood transfusion service readiness and its associated factors in health facilities providing blood transfusion services across Ethiopia: A secondary analysis of the 2018 Service Availability and Readiness Assessment (SARA) survey	Ethiopia	+	+	+	+		+
Gyedu et al. 2021 (74)	Assessing the appropriateness of blood transfusion among injured patients at a Ghanaian tertiary hospital: Time for clarity on the use of a scarce resource.	Ghana	**+**					
Jacobs et al. (2023) (44)	Survey of blood collection and transfusion practices among institutions in Africa	Rwanda, Senegal, Sudan, Tanzania, The Gambia, Uganda, Zambia, Zimbabwe, Botswana, Burundi, Cameroon, Cote d'Ivore, Egypt, Eswatini, Ethiopia, Ghana, Kenya, Lesotho, Liberia, Malawi, Mozambique, Nigeria	+			+		+
Jatau et al. 2022 (37)	Blood Transfusion Request and Utilization: The Trend in a Tertiary Health Care Centre in North Central Nigeria.	Nigeria	**+**					**+**
Kiguli et al. 2015 (57)	Anemia and blood transfusion in African children presenting to hospital with severe febrile illness	Kenya, Tanzania, Uganda	**+**			**+**		**+**
Mafirakureva et al. 2015 (72)	Profiles of blood and blood component transfusion recipients in Zimbabwe.	Zimbabwe			**+**			
Mafirakureva et al. 2016 (88)	The costs of producing a unit of blood in Zimbabwe.	Zimbabwe					**+**	
Mumo et al. 2023 (56)	Geographic accessibility and hospital competition for emergency blood transfusion services in Bungoma, Western Kenya.	Kenya	**+**			**+**		**+**
Musa et al. 2014 (36)	Pattern of blood transfusion request and utilization at a Nigerian University Teaching Hospital.	Nigeria	**+**			**+**		**+**
Nabwera et al. 2016 (54)	Pediatric blood transfusion practices at a regional referral hospital in Kenya	Kenya	**+**			**+**		**+**
Natukunda et al. 2010 (84)	Assessment of the clinical transfusion practice at a regional referral hospital in Uganda.	Uganda	**+**		**+**	**+**		**+**
Njolomole et al. 2022 (79)	Meeting demand—Obstetric hemorrhage and blood availability in Malawi, a qualitative study.	Malawi	**+**	**+**	**+**	**+**	**+**	**+**
Opoka et al. 2018 (69)	High rate of inappropriate blood transfusions in the management of children with severe anemia in Ugandan hospitals.	Uganda	**+**	**+**		**+**		**+**
Patidar et al. 2022 (30)	Management of blood transfusion services in low-resource countries	DRC, Ethiopia, Kenya, Morocco, Nigeria, Rwanda	**+**			**+**	**+**	**+**
Reggiani et al. 2020 (85)	Emergency pediatric blood transfusion practices in Mozambique.	Mozambique				**+**		**+**
Shari et al. 2017 (66)	Emergency blood transfusion practices among anemic children presenting to an urban emergency department of a tertiary hospital in Tanzania.	Tanzania	**+**			**+**		**+**
Tadeu and Geelhoed. 2016 (73)	“This thing of testing our blood is really very important”: a qualitative study of primary care laboratory services in Tete Province, Mozambique.	Mozambique	**+**	**+**		**+**	**+**	**+**
Thomas et al, 2017 (7)	Blood transfusion delay and outcome in county hospitals in Kenya.	Kenya	**+**					
Tort et al. 2015 (60)	Factors associated with postpartum hemorrhage maternal death in referral hospitals in Senegal and Mali: a cross-sectional epidemiological survey.	Senegal & Mali	**+**	**+**		**+**		
Uche et al. (2025) (43)	Assessment of Blood Transfusion Practices in Healthcare Institutions Across Abia State, Nigeria	Nigeria	+	+	+	+		+
Umar et al. (2024) (42)	Blood donation practices, processing and utilization of blood components in government tertiary hospitals in Nigeria: a multicentre cooperative study	Nigeria	+			+		
Weeber et al. 2018 (52)	Estimated injury-associated blood loss versus availability of emergency blood products at a district-level public hospital in Cape Town, South Africa.	South Africa	**+**		**+**	**+**	**+**	**+**
Wentzel et al. 2019 (53)	Fresh frozen plasma for on-demand hereditary angioedema treatment in South Africa and Iran.	South Africa	**+**			**+**		**+**

### Access to essential medicines

According to the WHO Health System Framework, access to essential medicines refers to the ready availability of affordable medicines to the public. As blood and blood products are captured in the WHO's Essential Medicines list, this indicates the importance of their access for all 12. Twenty-six (*n* = 26/33; 78%) (27, 30, 31, 36, 41–44, 52–54, 56, 57, 60, 63, 66, 67, 69, 73, 76, 78, 79, 83–86) studies discussed barriers related to a lack of or inadequate supply or use of blood and blood products in treatment, Table 3. Weeber et al. (2018) (52) in South Africa reported that the available blood in the emergency room for injury-associated hemorrhage might be sufficient in most cases, but markedly insufficient in the event of massive blood loss or for patients with other indications, particularly during weekends with twice the need for blood units. Natunkunda et al. (2010) (84) opined that 180/1674 (11%) of transfused patients who received additional blood transfusions within ≥48 h might have experienced blood transfusion shortages due to low blood stock. However, the over 3,887/218,564 (1.8%) unmet requested components are more telling of non-availability due to blood stockouts or lack of replacement donors, leading to delayed diagnosis, treatment, and contributing to high mortality (49,54,60). In Ethiopia, Fenta and colleagues found suboptimal blood transfusion readiness, with significant variation across regions. Overall, the authors found a mean readiness score of 4.5 out of 7, per the WHO Service Availability and Readiness Assessment guide (63).

### Leadership/governance

Effective leadership and governance ensure that strategic policy frameworks and accountability systems are in place to manage systems. Twenty-two (*n* = 22/33; 66%) (30, 31, 36, 37, 43, 44, 52–54, 56, 57, 63, 66, 67, 69, 73, 76, 79, 83–86) studies reported various leadership and governance-related barriers to blood and blood product access, such as poor adherence to national and WHO standards, lack of standardized transfusion protocol, lack of infrastructure, and poor transfusion management systems, Table 3. Reggiani et al. (2019) (85) found that 22% of transfusion recipients had Hb levels exceeding the WHO recommendations for severe anemia. Unfortunately, even children who met the WHO recommendations were not transfused. Beyond the poor adherence to recommended transfusion standards, some hospitals did not have standardized protocols and practices. This led to the clinicians using their judgment or disregarding laboratory test results for transfusion. In Uganda, authors reported the complete unavailability of hospital guidelines on the appropriate use of blood at one regional referral hospital (84). Also in Uganda, despite the availability of hospital guidelines, 52% of blood requests for children with severe anemia were sent solely on the clinician's judgment without laboratory confirmation of anemia (69). Lack of medical infrastructure, such as medical equipment, and the poor product forecasting and management of blood transfusion systems, led to blood shortages and undue delays (31, 73, 78). Furthermore, among 13 facilities investigated in Nigeria, none had a dedicated transfusion leadership committee 43. No studies were found which compared the leadership of central vs. hospital-based blood transfusion services, or detailed differences in leadership competencies between medical and financial executives.

### Financing

Financing involves mobilizing, accumulating, and allocating funds for health needs and purposes. In this study, eight (*n* = 8/33; 24%) (30, 31, 52, 73, 76, 79, 86, 88) papers discussed financial barriers to blood and blood product use, Table 3. However, most were focused on individual-level barriers, such as financial hardships and the inability to pay for blood services, and no studies were found which reported on the effect of risk sharing and health insurance on access to blood and blood products. In Uganda, parents or caregivers of children who had recently been transfused for severe anemia reported that costs of blood units were expensive at 30,000 shillings (approximately US$10) a unit and were often unavailable to purchase (76). Similarly, Tadeu and Geelhoed (2016) (73) reported that patients experienced financial hardships not only when purchasing blood components but also when traveling from one referral center to another. Costs of cross-matching, hiring staff, buying equipment, or collecting, analyzing, and storing blood products were additional challenges to health facilities. For example, Mafirakureva (2016) (88) reported an astronomically high cost of producing blood units (US$8.6 million) relative to the annual GDP of Zimbabwe. The overall cost of producing one safe unit of whole blood, RBCs, FFP, and PLTs was US$118.42, US$130.94, US$199.46, and US$76.09, respectively, constituting 12.4%, 13.7%, 20.9%, and 8.0%, respectively, of the country's annual GDP per capita of US$953). Checkley and colleagues (2019) in Uganda (86) further found that costs associated with machinery and maintenance for testing transfusion-transmitted infections constrained the ability to provide safe blood for patients.

### Health information systems

Health information systems refer to using data and information to enhance health-related decisions. Only eight (*n* = 8/33; 24%) (31, 43, 52, 72, 76, 79, 83, 84) papers discussed health information system-related challenges in this study, Table 3. The challenges centered on manual data collection and entry processes, leading to poor-quality data. In Zimbabwe, authors noted that blood bank registry data were collected manually. As a result, only 75% of discharge records were available for analysis (72). Corresponding findings were reported in Nigeria, where only six of the 31 participating hospitals had centrally administered blood banking systems to coordinate transfusions (31). Even when systems were available, logistical, and technical problems further complicated provider communication sharing, leading to poor decision-making and delayed care. In a qualitative study, the participants reported non-functional phone lines between the maternity department and the laboratory. Unfortunately, using WhatsApp to mitigate communication barriers worsened the situation as key providers were not included in the WhatsApp chat chain (79).

### Health workforce

The WHO Health Systems Framework describes the health workforce as individuals who engage in activities with a primary intent to enhance health. In this study, the health workforce refers to physicians, lab technicians, or other hospital staff. Only six (*n* = 6/33; 18%) (43, 60, 69, 73, 79, 83) studies reported barriers associated with the health workforce, Table 3. The primary barrier was the shortage of physicians or staff to conduct laboratory testing or other important clinical functions linked with blood services. In a qualitative study on blood transfusion management among children with severe anemia in Uganda, the authors reported low staffing levels, particularly qualified physicians in the evening shifts, which account for 48.5% of admissions (69). Even when over 80% of women in a maternity unit were seen and treated by a gynecologist-obstetrician, less than 50% of women were attended to by anesthesia staff and did not have access to an adult care unit. As expected, this study found a 45% decreased risk of maternal death in hospitals with a gynecologist-obstetrician on staff (60). Meanwhile, technicians' working hours were wasted in Tanzania due to unnecessary blood cross-matching, leading to a loss of over 73% of working hours during the study period of 171 days (83). In Nigeria, Uche and colleagues noted that no transfusion quality officers were identified across the 13 studies in one Nigerian state^43^.

### Facilitators of blood and blood component transfusion using the WHO health systems framework

Out of thirty-three articles that reported either barriers or facilitators, a total of thirteen articles (*n* = 13; 39%), Table 4, discussed facilitators of blood and blood product use. Of these, service delivery facilitators were reported in eight (*n* = 8/33; 24%) (7, 33, 36, 42, 52, 55, 57, 67, 78) studies and comprised the timely dispatch of blood using drones (unmanned aerial vehicles), prompt referral and appropriate transfusion. Five (*n* = 5/33; 15%) (7, 36, 50, 57, 78) articles highlighted access to essential medicine facilitators, such as reduced blood delivery times and cold-chain maintenance. Skilled health workforce (*n* = 2/33; 6% (57, 71); sound leadership/governance, which included policies on blood redistribution and quality management systems (*n* = 2/33; 6%) (30, 80); and health-information systems (*n* = 1/33; 3%) (80) facilitators were also reported.

**Table 4:** Facilitators of blood and blood product access using the WHO health system building blocks.

Author/Year	Title	Country	Service delivery	Health workforce	Health information systems	Access to essential medicines	Financing	Leadership/ Governance
Aliyu et al. 2017 (38)	Blood transfusion request pattern in a medical center in Northwestern Nigeria	Nigeria	**+**			**+**		
Kiguli et al. 2015 (57)	Anemia and blood transfusion in African children presenting to hospital with severe febrile illness	Kenya, Tanzania, Uganda	**+**					
Nabwera et al. 2016 (54)	Pediatric blood transfusion practices at a regional referral hospital in Kenya	Kenya	**+**					
Nisingizwe et al. 2022 (80)	Effect of unmanned aerial vehicle (drone) delivery on blood product delivery time and wastage in Rwanda: a retrospective, cross-sectional study and time series analysis.	Rwanda	**+**		**+**	**+**		**+**
Okoroiwu & Okafor 2018 (35)	Demographic characteristics of blood and blood components transfusion recipients and pattern of blood utilization in a tertiary health institution in southern Nigeria	Nigeria	**+**					
Opoka et al. 2018 (69)	High rate of inappropriate blood transfusions in the management of children with severe anemia in Ugandan hospitals.	Uganda	**+**					
Patidar et al. 2022 (30)	Management of blood transfusion services in low-resource countries.	DRC, Ethiopia, Kenya, Morocco, Nigeria, Rwanda						**+**
Tadeu and Geelhoed, 2016 (73)	“This thing of testing our blood is really very important”: a qualitative study of primary care laboratory services in Tete Province, Mozambique.	Mozambique		**+**				
Thomas et al. 2017 (7)	Blood transfusion delay and outcome in county hospitals in Kenya.	Kenya	**+**			**+**		
Tort et al. 2015 (60)	Factors associated with postpartum hemorrhage maternal death in referral hospitals in Senegal and Mali: a cross-sectional epidemiological survey.	Senegal Mali	**+**	**+**		**+**		
Umar et al. (2024) (42)	Blood donation practices, processing and utilization of blood components in government tertiary hospitals in Nigeria: a multicentre cooperative study	Nigeria						+
Weeber et al. 2018 (52)	Estimated injury-associated blood loss versus availability of emergency blood products at a district-level public hospital in Cape Town, South Africa.	South Africa				**+**		

## Discussion

Challenges in access to safe blood and blood products in sub-Saharan Africa (SSA) have long persisted. These require detailed interrogation of national blood systems and practices to understand why despite decades of the existence of blood services in SSA, these gaps continue to challenge safe blood availability and use. We conducted a review of 65 studies representing 18 SSA countries, assessed blood transfusion practices and patterns, and evaluated barriers and facilitators of blood and blood product use on the continent.

Whole blood (WB) was an important source for blood transfusions in 76% of the locations studied, and fresh frozen plasma (FFP) was the most utilized blood component. As plasmapheresis is largely underutilized in African countries, these FFP are most likely derived from the splitting of WB into components post-donation 2. Despite blood components being the conventional method for targeted patient therapy, the capital-intensive nature of the investment in blood component production and fractionation hinders many SSA countries (88–91).

Pediatrics reported the highest prevalence of transfusion, mostly for anemia-related conditions such as malaria, sickle cell disease, and severe acute malnutrition. Transfusion in obstetrics, especially peripartum hemorrhage, was next, in alignment with other studies (29, 90–92). It is notable that in SSA, the proportions of transfused SCA patients have been reported as 90% (93). Malaria and anemia are major contributors to child mortality in SSA, with rates of 70 per 1,000 live births, which is nearly twice the global average and twenty times the average for children in Western Europe (94). Despite global declines of up to 40% in maternal deaths between 2000 and 2020, rates on the continent remain abysmal, contributing nearly 70% (95) of global maternal deaths.

Leveraging the WHO health system framework, we analyzed blood transfusion systems. Across the framework's six building blocks, limited service delivery and access to blood as an essential medicine were reportedly the major obstacles to access in SSA. These barriers, which comprised stock-outs, prolonged intervals before transfusion, and inappropriate transfusion practices, could be attributable to poor care coverage and insufficient training (96, 97). Other barriers included poor leadership and governance, inadequate financing, manualized health information systems, and health workforce shortages.

## Policy and public health implications

The service delivery and accessibility challenges in SSA's access to blood are major issues of concern as they widen existing health inequities. Innovative reforms in cold and supply chain mechanisms, such as drone technology for delivery to remote locations, community-based transport programs, ambulance and transportation infrastructure, and enhancing referral systems, would be critical (80, 97, 98). Safe blood is an essential and potentially life-saving commodity; hence, considering the burden of anemia-related conditions and post-partum bleeding in SSA, ensuring timely access would be critical. Using drone delivery in Rwanda was found to shorten delivery times by 79–98 min and reduce blood expiry by 67% (80). These gains can be translated to shortened intervals between prescription and transfusion due to logistics improvements, with commensurate improvements in child and maternal death rates from malaria, anemia, and post-partum bleeding (7, 9, 10, 26, 27, 37, 60, 78, 79). Regardless of the immense gains reported from Rwanda, there is little evidence of substantial uptake of drone technology in other African countries, perhaps owing to its implementation being capital-intensive.

Furthermore, addressing stock-outs and shortages of blood would require enhancing blood donor pools in communities. There is a massive dearth of voluntary donors in SSA, which greatly impedes the effectiveness of transfusion services (99–102). In the WHO African region, the average number of blood donations is less than 6 units per 1,000 population, and even as low as two units per 1,000 population in countries such as Cameroon and Eritrea (2, 99, 100). Community-based interventions promoting blood donations in particular sociocultural groups, such as the use of language, involvement of community members in donor recruitment, or the use of culturally relevant demonstrations, have been reportedly successful and could be explored (101, 103).

The absence of effective leadership and governance of blood services was the third most cited barrier amongst the building blocks, and being multi-faceted has implications for several other blocks. For instance, under the leadership and governance block, adherence to relevant transfusion standards and protocols would prevent inappropriate transfusions and wastages identified in the service delivery block (67, 83, 84). Additionally, sound governance of blood services would strengthen blood supplies through timely tracking of donated blood using health information systems (80). None of the included studies explored the importance of leadership competencies and organization type in the effectiveness of blood services. However, as organization and oversight are vital to any blood safety infrastructure (102), they need to be examined in future research.

Despite global attention and progress towards UHC, low levels of public financing in total health expenditure remain a challenge for many LMICs, with high OOP spending, and sub-optimal budgetary allocations to health (104). High blood production costs were financial barriers reported in this study (86, 88). Although costs may increase if more technologically advanced blood production and safety measures are implemented, adopting blood management programs would minimize inappropriate transfusions and blood wastage and reduce overall costs (105). Moreover, this could translate to reduced costs of care and minimized financial hardships for patients. No single study reported financing as a facilitator of blood and blood product access and use. This particularly highlights the enormous financing gap for safe blood services in SSA. Countries should endeavor to include blood and blood products as listed in the WHO essential medicines list in national and sub-national health services packages for health insurance reimbursement. Thus, significantly reducing the burden of OOP payments. In Nigeria, the Basic Minimum Package of Health Care Services (BMPHS) and Emergency Medical Treatment (EMT) are designed to cover this but remain largely under-utilized due to sub-national failures to meet eligibility criteria for infrastructure and accountability (106, 107). A financing challenge across several SSA countries is the over-reliance on external funding, which weakens the delivery and sustainability of health services (108–111). Between 2004 and 2016, significant financial support and technical assistance of about US$468 million was given to 25 SSA countries from the U.S. President's Emergency Plan for AIDS Relief (PEPFAR) to strengthen national blood transfusion services (NBTS) and improve blood safety and availability (112). However, following PEPFAR's exit from these countries, there is insufficient evidence of sustained national investments in blood services to build on that assistance.

Cost recovery for blood products was reportedly highest in East and Southern Africa, with the highest funding cost recovery at 83.8% compared to 9% in West Africa, indicating a significant cost burden for households and national governments (86, 112). Public-private partnerships could alleviate this cost burden, especially in community engagement, blood donor recruitment and retention, and screening, processing, and cold-chain maintenance (113). Exploring East and Southern African cost recovery mechanisms is worth exploring for cross-national learning.

Despite existing health workforce shortages in SSA (114), this block was interestingly the least cited by researchers. While African Member States, including Nigeria, have prioritized boosting the health workforce (115), these are medium-to-long terms strategies. Therefore, the strengthening of blood transfusion services in SSA would largely rely on the more short-to-medium term strategies earlier listed.

## Strength and limitations

This study's use of the WHO health system framework to understand the barriers and facilitators to blood product access and use in SSA offers fresh insights. Using a robust search strategy, eighteen out of the forty-nine countries in SSA were included for analysis. However, this study had several limitations. First, only articles in English were included for analysis. Therefore, findings may not be applicable throughout SSA, particularly in Francophone or Lusophone countries. Although several countries were represented in this study, the findings cannot be generalized due to cultural, social, political, and economic differences. Also, some critical components of blood safety practices are not overtly included in the WHO Framework. These include health workforce issues such as professional education and training and are therefore worth considering in future explorations of the relevance of this framework to blood safety in SSA. Finally, this study covered a large period, spanning two decades, with such a vast timeline presenting a likely limitation to the findings. However, we had anticipated observing changing trends over the course of the years between the establishment of organized blood services in much of SSA in 2005 and the formal introduction of the framework by WHO in 2007 (18, 19, 112, 116). Regardless, this study adds to the body of literature indicating potential areas of intervention to strengthen blood services in SSA.

## Conclusion

We investigated blood transfusion practices, patterns, and indications; and used the WHO health system building blocks to determine barriers and facilitators to blood and blood product access in SSA. Anemia-related conditions such as malaria, sickle cell disease, severe acute malnutrition, and peripartum hemorrhage were conditions that most frequently required blood transfusion, with whole blood being the most used product. Delayed transfusion, untimely referrals, inappropriate transfusion practices, low transfusion rates, blood stock-outs, and poor leadership and governance systems in blood services were the most prevalent.

This study revealed the use of mainly whole blood instead of blood components in managing severe anemia in children and peripartum hemorrhage. Findings from the systems assessment of barriers and facilitators of blood and blood product use point to the need for policymakers, managers of blood services, and transfusion professionals to deliberately conceptualize strategies to tackle bottlenecks to timely, safe blood supply. Shortened blood dispatch times, enablement of prompt referrals, and appropriate transfusion practice need to be prioritized to enhance the ready availability of safe blood in SSA. Despite its potential, the innovative use of drone technology in Rwanda to boost blood supply chains has not been scaled up to other parts of the continent and is worth emulating. Surprisingly, none of the papers reviewed highlighted financing or the effect of leadership competencies or organization systems as facilitators of using safe blood. Additionally, despite increasing global emphasis on risk sharing and universal health coverage, no research has demonstrated its potential for financing blood services. This reveals clear gaps in research that need to be explored in blood transfusion services in SSA.

Health systems in SSA are fragile, and an enormous burden of high blood-need conditions prevails in the region. Therefore, given her vulnerability to threats, namely terrorism, conflict, and infectious diseases, strengthening blood systems would help address existing challenges in access to safe blood and improve health outcomes, especially for women and children.

## Data Availability

The original contributions presented in the study are included in the article/Supplementary Material, and further inquiries can be directed to the corresponding author.
