# Supplementary material for: Facilitators and barriers to blood and blood product accessibility and use in sub-Saharan Africa: a systematic review
Source: Front Health Serv. 2026 Jun 12;6:1837188. doi: 10.3389/frhs.2026.1837188 (PMC13303813; doi:10.3389/frhs.2026.1837188)
Supplement: Supplementary file 1 [file Supplementaryfile1.docx]

**Title:** Facilitators and Barriers to Blood and Blood Product Accessibility and Use in sub-Saharan Africa: A Systematic Review

**Appendix 1**

**Data Source and Search Strategy**

Literature was searched from Web of Science, MEDLINE, PubMed, PsycINFO, Google Scholar, and Global Health databases. A thorough manual search was also conducted in the references of the identified studies. To ensure a comprehensive and unbiased investigation, a preliminary search was conducted on PubMed by scrutinizing the article title, abstracts, and keywords, which was further adapted and modified for this current review. The search strategy was limited to articles published in English between 2005 and September 2023. An updated search was conducted between 2023 and February 2026. Moreover, the timeline was selected because in 2005, WHO Member States committed to universal health care coverage, paving the way for stronger national policies and legislature to bolster access to safe blood and blood products in line with the U.S. President’s Emergency Plan for AIDS Relief (PEPFAR) which led to the formation of national blood blanks and services across Africa. The search term used the “AND” and “OR” Boolean operators to separate keywords consisting of Medical Subject Headings (MeSH). The keywords used included: “blood transfusion,” OR blood products,” “plasma,” OR “red blood cells,” OR “platelets,” OR “fresh frozen plasma,” “cryoprecipitate,” OR “access,” OR “utilization,” AND “"Angola" OR "Benin" OR "Botswana" OR "Burkina Faso" OR "Burundi" OR "Cameroon" OR "Cape Verde" OR "Central African Republic" OR "Chad" OR "Comoros" OR "Congo" OR "Democratic Republic of Congo" OR "Cote d'Ivoire" OR "Djibouti" OR "Equatorial Guinea" OR "Eritrea" OR "Ethiopia" OR "Gabon" OR "Gambia" OR "Ghana" OR "Guinea" OR "Guinea‐Bissau" OR "Kenya" OR "Lesotho" OR "Liberia" OR "Madagascar" OR "Malawi" OR "Mali" OR "Mauritania" OR "Mauritius" OR "Mozambique" OR "Namibia" OR "Niger" OR "Nigeria" OR "Rwanda" OR "Sao Tome" OR "Senegal" OR "Seychelles" OR "Sierra Leone" OR "Somalia" OR "South Africa" OR "South Sudan" OR "Sudan" OR "Swaziland" OR "eSwatini" OR "Tanzania" OR "Togo" OR "Uganda" OR "Zambia" OR "Zimbabwe"

**Study Selection**

Following a comprehensive and robust three-stage screening: title/abstract, full-text, and extraction, studies that did not meet the studies inclusion and exclusion criteria alongside the duplicates were removed. The inclusion and exclusion criteria were formulated in alignment with the research objectives. All included studies were screened using Covidence software. Three independent reviewers evaluated and screened each article's eligibility for inclusion or exclusion per the study’s objectives, with the fifth reviewer providing a consensus where a tiebreaker was required for the resolution of any conflict. A study protocol was then developed to guide the screening process. After each reviewer extracted the data, they compared their records, and any differences were resolved by consensus before articles were deemed eligible to be included for final review. Moreover, the search strategy and the article's eligibility were based on the Population, Intervention, Comparison, and Outcome (PICO) model for clinical questions to accurately frame and answer the research questions.

**Appendix 1 Table 1: PICO Model for Clinical Questions**

| **Criteria** | **Determinant** |
| --- | --- |
| Population | Patients across the life course |
| Intervention | All studies (quantitative & qualitative) on blood and blood product in sub-Saharan Africa |
| Comparison | Where applicable for intervention or case-control |
| Outcome | Relevant outcomes related to blood services |

**Inclusion and Exclusion Criteria**

The table below shows the inclusion and exclusion criteria used to screen and extract studies included for review.

**Appendix 1 Table 2: Inclusion and Exclusion Criteria**

| Inclusion Criteria | Exclusion Criteria |
| --- | --- |
| 1. Original research articles reporting blood and blood product transfusion, access, or utilization in sub-Saharan Africa | 1. Articles that did not report blood and blood product transfusion, access, or utilization (e.g., blood donor programs). |
| 1. Articles published in peer-reviewed journals with any study design addressing the study question. | 1. Non-peer-reviewed articles, irrespective of the study design and geographical location. |
| 1. Articles published in English Language. | 1. Articles in languages other than English |
| 1. Articles published between 2005 and September 2023. | 1. Articles not conducted in sub-Saharan African countries. |
| 1. Articles involving all patient demographics and health conditions addressing the research question. |  |

**Appendix 2: Quality Appraisal and Risk of Bias Assessment**

Methodological quality assessment was done on all included studies using a modified Joanna Briggs Checklist. Forty-four (68%) of the studies had a high-quality evaluation, with over 76 and 100% of each appraisal criterion scoring “yes.” The remaining eleven studies had an average quality of 50-57%, scoring “yes” in each appraisal category. None of the studies attained lower than 50% “yeses,” thereby showing a minimal risk of bias in the overall evidence across all study types.

**Appendix 2 Table 1: Study Quality Appraisal**

| **Criteria** | 1.     Were the criteria for inclusion in the sample clearly defined? | 2.     Were the study subjects and the setting described in detail? | 3.     Was the exposure measured in a valid and reliable way? | 4.     Were objective, standard criteria used for measurement of the condition? | 5.     Were confounding factors identified? | 6.     Were strategies to deal with confounding factors stated? | 7.     Were the outcomes measured in a valid and reliable way? | 8.     Was appropriate statistical analysis used? | 9.     Were the two groups similar and recruited from the same population? | 10.  Were the exposures measured similarly to assign people to both exposed and unexposed groups? | 11.  Was the exposure measured in a valid  and reliable way? | 12.  Was the follow up time reported and sufficient to be long enough for outcomes to occur? | 13.  Were the groups comparable other than the disease in cases or the presence of absence of disease in controls? | 14.  Were cases and controls matched appropriately? | 15.  Were the same criteria used for identification of cases and controls? | 16.  Was the exposure period of interest long enough to be meaningful? |  |  |
| --- | --- | --- | --- | --- | --- | --- | --- | --- | --- | --- | --- | --- | --- | --- | --- | --- | --- | --- |
| **Author:**  Ahmed et al  **Year:** 2019 | Y | Y | Y | Y | Y | N | Y | Y | N/A | N/A | N/A | N/A | N/A | N/A | N/A | N/A |  |  |
| **Author:** Akech et al  **Year:** 2008 | Y | Y | Y | Y | Y | N | Y | Y | N/A | N/A | N/A | N/A | N/A | N/A | N/A | N/A |  |  |
| **Author:** Akingbola & Bello  **Year:** 2016 | Y | Y | Y | Y | Y | Y | Y | Y | N/A | N/A | N/A | N/A | N/A | N/A | N/A | N/A |  |  |
| **Author:** Akinlusi et al  **Year:**2018 | Y | Y | Y | Y | N | N/A | Y | Y | Y | Y | Y | N/A | N/A | N/A | N/A | N/A |  |  |
| **Author:** Akoko & Joseph  **Year:**2015 | Y | Y | Y | Y | N | N/A | Y | Y | Y | Y | Y | N/A | N/A | N/A | N/A | N/A |  |  |
| **Author:** Akpa et al.  **Year:**2022 | Y | Y | Y | Y | Y | Y | Y | Y | N/A | N/A | N/A | N/A | Y | Y | Y | Y |  |  |
| **Author:** Akwiwu et al. Year: 2024 | Y | Y | Y | Y | Y | Y | NA | NA | NA | NA | NA | NA | NA | NA | NA | NA |  |  |
| **Author:** Aliyu et al  **Year:** 2017 | Y | Y | Y | Y | Y | Y | Y | Y | N/A | N/A | N/A | N/A | N/A | N/A | N/A | N/A |  |  |
| **Author:** Amadi et al  **Year:**2023 | Y | Y | Y | Y | Y | Y | Y | Y | N/A | N/A | N/A | N/A | N/A | N/A | N/A | N/A |  |  |
| **Author:** Assennato et al  **Year:** 2018 | Y | Y | Y | Y | Y | N | Y | Y | N/A | N/A | N/A | N/A | N | N | N | N/A |  |  |
| **Author: Asuquo et al.**  **Year: 2025** | Y | Y | NA | Y | Y | N | N | Y | N/A | N/A | N/A | N/A | N | N | N | N/A |  |  |
| **Author: Bassey et al.**  **Year: 2024** | Y | Y | Y | Y | N/A | Y | Y | Y | N | N | N/A | N/A | N/A | N/A | N/A | N/A |  |  |
| **Author:** Birhan & Asfaw  **Year:** 2019 | Y | Y | Y | Y | Y | Y | Y | Y | N/A | N/A | N/A | N/A | N/A | N/A | N/A | N/A |  |  |
| **Author:** Bloch et al  **Year:** 2018 | Y | Y | Y | Y | Y | Y | Y | Y | N/A | N/A | N/A | N/A | Y | Y | Y | Y |  |  |
| **Author:** Bolton et al  **Year:** 2021 | Y | Y | Y | Y | Y | Y | Y | Y | N/A | N/A | N/A | N/A | N/A | N/A | N/A | N/A |  |  |
| **Author:** Bugge et al  **Year:** 2013 | Y | Y | Y | Y | Y | Y | Y | Y | N/A | N/A | N/A | N/A | N/A | N/A | N/A | N/A |  |  |
| **Author:** Chansa et al.  **Year:** 2014 | N/A | N/A | N/A | N/A | N/A | N/A | N/A | N/A | N/A | N/A | N/A | N/A | N/A | N/A | N/A | N/A |  |  |
| **Author:** Checkley et al  **Year:**2019 | Y | Y | Y | Y | N | N | Y | Y | N/A | N/A | N/A | N/A | N/A | N/A | N/A | N/A |  |  |
| **Author: Chiabi et al.**  **Year: 2024** | Y | Y | Y | Y | N | N | N/A | N/A | N/A | N/A | N/A | N/A | N/A | N/A | N/A | Y |  |  |
| **Author:** Dhabangi et al  **Year:**2019 | N/A | N/A | N/A | N/A | N/A | N/A | N/A | N/A | N/A | N/A | N/A | N/A | N/A | N/A | N/A | N/A |  |  |
| **Author:** Diaku-Akinwumi et al.  **Year:** 2016 | N | Y | Y | Y | Y | N | Y | Y | N/A | N/A | N/A | N/A | N/A | N/A | N/A | N/A |  |  |
| **Author:** Drammeh et al  **Year:** 2018 | Y | Y | Y | Y | Y | Y | Y | Y | N/A | N/A | N/A | N/A | N/A | N/A | N/A | N/A |  |  |
| **Author: Efobi et al.**  **Year: 2021** | Y | Y | Y | Y | Y | Y | Y | Y | N/A | N/A | N/A | N/A | N/A | N/A | N/A | N/A |  |  |
| **Author:** Eyelade et al  **Year**:2015 | Y | Y | Y | Y | Y | Y | Y | Y | N/A | N/A | N/A | N/A | N/A | N/A | N/A | N/A |  |  |
| **Fenta et al.**  **Year 2024** | Y | Y | Y | Y | Y | Y | Y | Y | N/A | N/A | N/A | N/A | Y | Y | Y | Y |  |  |
| **Author:** George et al.  **Year:** 2022 | Y | Y | Y | Y | Y | Y | Y | Y | N/A | N/A | N/A | N/A | Y | Y | Y | Y |  |  |
| **Author:** Gyedu et al  **Year:** 2021 | Y | Y | Y | Y | Y | Y | Y | Y | N/A | N/A | N/A | N/A | N/A | N/A | N/A | N/A |  |  |
| **Author: Ijah et al.**  **Year: 2024** | Y | N | Y | Y | N/A | N/A | N/A | Y | N/A | N/A | N/A | N/A | N/A | N/A | N/A | Y |  |  |
| **Author: Jacobs et al.**  **Year: 2023** | Y | Y | Y | Y | Y | Y | Y | Y | N/A | N/A | N/A | N/A | N/A | N/A | N/A | N/A |  |  |
| **Author:** Jatau et al  **Year:** 2022 | Y | N | Y | Y | N/A | N/A | N/A | Y | N/A | N/A | N/A | N/A | N/A | N/A | N/A | N/A |  |  |
| **Author:** Keating et al  **Year:** 2021 | Y | Y | Y | Y | Y | Y | Y | Y | Y | Y | Y | Y | N/A | N/A | N/A | N/A |  |  |
| **Author**: Kiguli et al  **Year:** 2015 | Y | Y | Y | Y | Y | Y | Y | Y | N/A | N/A | N/A | N/A | N/A | N/A | N/A | N/A |  |  |
| **Author: Kintieti et al.**  **Year: 2026** | Y | Y | Y | Y | Y | Y | Y | Y | Y | Y | Y | Y | N/A | N/A | N/A | N/A |  |  |
| **Author:**  Mafirakureva et al  **Year:** 2015 | Y | Y | Y | Y | Y | Y | Y | Y | N/A | N/A | N/A | N/A | N/A | N/A | N/A | N/A |  |  |
| **Author:**  Mafirakureva  **Year:**2015 | Y | Y | N/A | N/A | N/A | N/A | N/A | Y | N/A | N/A | N/A | N/A | N/A | N/A | N/A | N/A |  |  |
| **Author:**  Mandar et al  **Year:**2022 | Y | Y | Y | Y | Y | Y | Y | Y | N/A | N/A | N/A | N/A | N/A | N/A | N/A | N/A |  |  |
| **Author:**  Morris et al  **Year:**2019 | Y | Y | Y | Y | Y | N | Y | Y | N/A | N/A | N/A | N/A | N/A | N/A | N/A | N/A |  |  |
| **Author:**  Mumo et al  **Year:**2023 | Y | Y | Y | Y | Y | N | Y | Y | N/A | N/A | N/A | N/A | N/A | N/A | N/A | N/A |  |  |
| **Author:** Musa et al  **Year:**2023 | N | Y | Y | Y | N | N | Y | N | N/A | N/A | N/A | N/A | N/A | N/A | N/A | N/A |  |  |
| **Author:** Nabwera et al  **Year:**  **2016** | Y | Y | Y | Y | Y | Y | Y | Y | N/A | N/A | N/A | N/A | N/A | N/A | N/A | N/A |  |  |
| **Author:** Natukunda et al  **Year:** 2010 | Y | Y | Y | Y | Y | Y | Y | Y | N/A | N/A | N/A | N/A | N/A | N/A | N/A | N/A |  |  |
| **Author:** Nisingizwe et al  **Year:** 2022 | N | Y | Y | Y | Y | Y | Y | Y | N/A | N/A | N/A | N/A | N/A | N/A | N/A | N/A |  |  |
| **Author:** Njolomole  **Year:** 2022 | N/A | N/A | N/A | N/A | N/A | N/A | N/A | N/A | N/A | N/A | N/A | N/A | N/A | N/A | N/A | N/A |  |  |
| **Author:** Nwafor et al  **Year:** 2018 | Y | Y | Y | Y | N | Y | N | Y | N/A | N/A | N/A | N/A | N/A | N/A | N/A | N/A |  |  |
| **Author: Linstron et al. (2024)** | Y | Y | Y | Y | Y | Y | Y | Y | Y | Y | Y | Y | Y | N/A | N/A | Y |  |  |
| **Author:** Okoroiwu & Okafor  **Year:** 2018 | Y | Y | Y | Y | Y | Y | Y | N | N/A | N/A | N/A | N/A | N/A | N/A | N/A | N/A |  |  |
| **Author:** Olupot-Olupot et al  **Year:** 2017 | Y | Y | Y | Y | Y | Y | Y | Y | N/A | N/A | N/A | N/A | N | Y | Y | Y |  |  |
| **Author:** Opoka et al  **Year:** 2018 | Y | Y | Y | Y | Y | Y | Y | Y | N/A | N/A | N/A | N/A | N/A | N/A | N/A | N/A |  |  |
| **Author:**  Oreh et al  **Year:** 2022 | N | Y | Y | Y | Y | Y | Y | Y | N/A | N/A | N/A | N/A | N/A | N/A | N/A | N/A |  |  |
| **Author:** Patidar et al  **Year:** 2022 | Y | Y | Y | Y | N | N | Y | N | N/A | N/A | N/A | N/A | N/A | N/A | N/A | N/A |  |  |
| **Author:** Pitman et al  **Year:** 2015 | N | Y | Y | Y | Y | Y | Y | Y | N/A | N/A | N/A | N/A | N/A | N/A | N/A | N/A |  |  |
| **Author:** Ramtohul et al  **Year:** 2022 | Y | Y | Y | Y | Y | Y | Y | Y | N/A | N/A | N/A | N/A | N/A | N/A | N/A | N/A |  |  |
| **Author:** Reggiani et al  **Year:** 2020 | Y | Y | Y | Y | Y | Y | Y | Y | N/A | N/A | N/A | N/A | N/A | N/A | N/A | N/A |  |  |
| **Author:** Salami et al  **Year:** 2022 | Y | Y | Y | Y | Y | Y | Y | Y | N/A | N/A | N/A | N/A | N/A | N/A | N/A | N/A |  |  |
| **Author:** Sawadogo et al  **Year:** 2020 | Y | Y | Y | Y | Y | Y | Y | Y | N/A | N/A | N/A | N/A | N/A | N/A | N/A | N/A |  |  |
| **Author:** Shari et al  **Year:** 2017 | Y | Y | Y | Y | Y | Y | Y | Y | N/A | N/A | N/A | N/A | N/A | N/A | N/A | N/A |  |  |
| **Author:** Tadeuy & Geelhoed  **Year:** 2016 | Y | Y | N/A | N/A | N/A | N/A | Y | N/A | N/A | N/A | N/A | N/A | Y | N/A | N/A | N/A |  |  |
| **Author:** Tewabe et al  **Year:** 2022 | Y | Y | Y | Y | Y | Y | Y | Y | N/A | N/A | N/A | N/A | N/A | N/A | N/A | N/A |  |  |
| **Author:**  Thomas et al.  **Year:** 2017 | Y | Y | Y | Y | Y | Y | Y | Y | N/A | N/A | N/A | N/A | N/A | N/A | N/A | N/A |  |  |
| **Author:**  Tort et al  **Year:** 2015 | Y | Y | Y | Y | Y | Y | Y | Y | N/A | N/A | N/A | N/A | N/A | N/A | N/A | N/A |  |  |
| **Author:** Tsime et al  **Year:** 2016 | Y | Y | Y | Y | Y | Y | Y | Y | N/A | N/A | N/A | N/A | N/A | N/A | N/A | N/A |  |  |
| **Author: Uche et al.**  **Year: 2025** | Y | Y | Y | Y | Y | Y | N/A | N/A | N/A | N/A | N/A | N/A | N/A | N/A | N/A | N/A |  |  |
| **Author: Umar et al.**  **Year 2024** | Y | Y | Y | Y | Y | Y | N/A | N/A |  | Y | Y | Y | N/A | N/A | N/A | N/A |  |  |
| **Author:** Weeber et al  **Year:** 2018 | Y | Y | Y | Y | Y | Y | Y | Y | N/A | N/A | N/A | N/A | N/A | N/A | N/A | N/A |  |  |
| **Author:** Wentzel et al  **Year:** 2019 | N | Y | Y | Y | Y | Y | N | Y | N | N/A | N/A | N | N/A | N/A | N/A | N/A |  |  |
